# The unexpected influence of aryl substituents in *N*-aryl-3-oxobutanamides on the behavior of their multicomponent reactions with 5-amino-3-methylisoxazole and salicylaldehyde

**DOI:** 10.3762/bjoc.10.320

**Published:** 2014-12-17

**Authors:** Volodymyr V Tkachenko, Elena A Muravyova, Sergey M Desenko, Oleg V Shishkin, Svetlana V Shishkina, Dmytro O Sysoiev, Thomas J J Müller, Valentin A Chebanov

**Affiliations:** 1 Division of Chemistry of Functional Materials, SSI “Institute for Single Crystals” NAS of Ukraine, Lenin Ave. 60, Kharkiv 61001, Ukraine; 2 Faculty of Chemistry, V. N. Karazin Kharkiv National University, Svobody sq. 4, Kharkiv 61022, Ukraine; 3 Fachbereich Chemie, University of Konstanz, Fach M-720, Universitaetsstrasse 10, D-78457 Konstanz, Germany; 4 Institut für Organische Chemie und Makromolekulare Chemie, Heinrich-Heine-Universität Düsseldorf, Universitätsstraße 1, D-40225 Düsseldorf, Germany

**Keywords:** 5-amino-3-methylisoxazole, catalysis, chemoselectivity, heterocycle, multicomponent reaction, ultrasonication, salicylaldehyde

## Abstract

The switchable three-component reactions of 5-amino-3-methylisoxazole, salicylaldehyde and *N*-aryl-3-oxobutanamides under different conditions were studied and discussed. The unexpected influence of the aryl substituent in *N*-aryl-3-oxobutanamides on the behavior of the reaction was discovered. The key influence of ultrasonication and Lewis acid catalysts led to an established protocol to selectively obtain two or three types of heterocyclic scaffolds depending on the substituent in the *N*-aryl moiety.

## Introduction

Generally, considerable interest in heterocyclic compounds is due to their key role in biological processes in nature and biological activity. In particular, condensed heterocycles bearing a hydroxyaryl group as well as tricyclic nitrogen-containing heterocycles derived from salicylaldehyde have been reported as anticancer [1], antihypertensive agents [2], neuropeptide Y antagonists [3], and calcium channel blockers [4]. Fused azoloazines containing carboxamide substituents also exhibit a broad spectrum of biological activity [5–6], which led to choose acetoacetamides as perspective methylene-active compounds for further studies of multicomponent reactions.

**Scheme 1:**
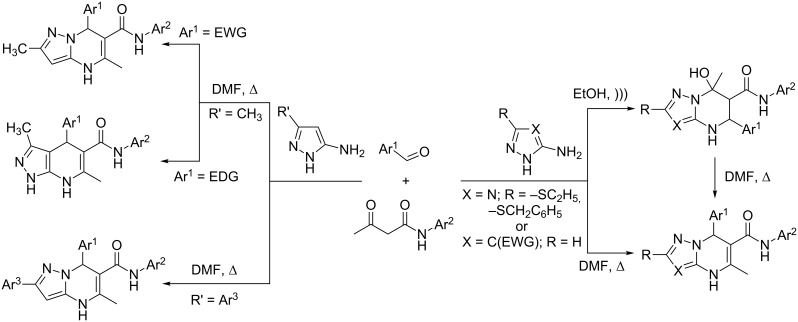
Some three-component reactions involving *N*-aryl-3-oxobutanamides.

The interactions of 3-oxobutanamides with aldehydes and a number of aminoazoles, namely 3-amino-1,2,4-triazoles [7–11], 5-aminotetrazole [9], 5-aminopyrazoles [11], and 5-amino-1,2,3-triazole [12] have previously been investigated. In some cases when the reaction could proceed in two alternative pathways the conditions enabling to control the interaction direction were determined, which made it possible to obtain the desired azoloazine with high chemo- and regioselectivity [11,13–15]. In particular, three-component heterocyclizations involving 3-amino-1,2,4-triazoles or 4-substituted 5-aminopyrazoles yielded either 4,5,6,7-tetrahydroazolo[1,5-*a*]pyrimidine-6-carboxamides under ultrasonication at room temperature (kinetic control) or 4,7-dihydroazolo[1,5-*a*]pyrimidine-6-carboxamides at reﬂux in an applicable solvent (thermodynamic control), respectively (Scheme 1). The behavior of the reaction of 5-aminopyrazoles containing substituents in the position 3 is influenced by the structure of aminoazoles and aldehydes, giving rise either to pyrazolopyridine or pyrazolopyrimidine heterocycles. In the authors' opinion, the different outcomes in three-component reactions involving 3-methyl- or 3-aryl-substituted 5-aminopyrazoles can mainly be put down to the steric substituent effect [11].

It is noteworthy to mention that studies on multicomponent heterocyclizations involving different N-containing polynucleophiles and salicylaldehyde are of particular interest due to the bifunctional reactivity of the latter component. The presence of both electrophilic and nucleophilic reaction centers in salicylaldehyde enables condensations to proceed in various possible directions. The direction control would open pathways for selectively obtaining different classes of heterocyclic compounds [16–20]. Simultaneously, the selectivity will inevitably be dictated by the variation of the reaction conditions [17–18].

**Scheme 2:**
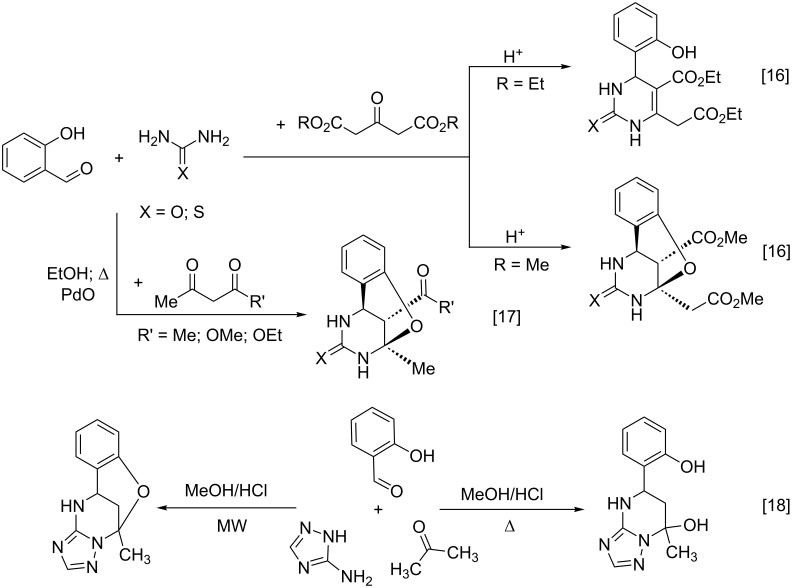
Some Biginelli-type three-component condensations with salicylaldehyde.

Thus, Světlik et al. studied the Biginelli-type condensation of 2-hydroxybenzaldehyde with urea (thiourea) and dialkyl acetone-1,3-dicarboxylates as active methylene components [16]. Unexpectedly, the reaction with salicylaldehyde furnished two different products depending on the ester alkyl group (Scheme 2). Obtaining of two classes of compounds was found to originate from steric influence rendered by the alkyl moiety of the ester group in the active methylene species. Inspired by Světlik’s studies, Jing et al. [17] developed an efficient method for the synthesis of oxygen-bridged pyrimidine tricyclic derivatives from salicylaldehyde, various dicarbonyl compounds, and urea (thiourea) using PdO as a catalyst (Scheme 2).

Heterocyclizations involving both, salicylaldehyde and aminoazoles, are intriguing as well. Thus, for example in the course of the study on the application of 3-amino-1,2,4-triazole and salicylaldehydes in a Biginelli-like three-component condensation, two alternative outcomes were observed by Gorobets et al. [18]. When the condensation with acetone was performed in methanol with catalytic amounts of HCl under reflux conditions, a tetrahydrotriazolo[1,5-*a*]pyrimidine derivative was obtained, while under microwave irradiation the substituted oxygen-bridged triazolo[1,5-*c*][1,3,5]benzoxadiazocine was formed (Scheme 2).

Interesting results were also described by Světlik and Kettmann [19]. In the case of a three-component interaction of aminoazole, salicylaldehyde, and methyl acetoacetate in refluxing ethanol in the presence of hydrochloric acid, instead of oxygen-bridged compounds oxadiazaspiranes were isolated. On the other hand, Umarov and coworkers [20] have synthesized by condensation of 5-ethyl-1,3,4-thiadiazol-2-amine, salicylaldehyde, and pentane-2,4-dione in refluxing ethanol a representative of another heterocyclic class, namely thiadiazolylaminochromane.

Here we disclose our recent findings of three-component heterocyclizations involving 5-amino-3-methylisoxazole, salicylaldehyde and *N*-aryl-3-oxobutanamides that were found to differ from similar reactions of cyclic CH acids [13].

## Results and Discussion

**Scheme 3:**
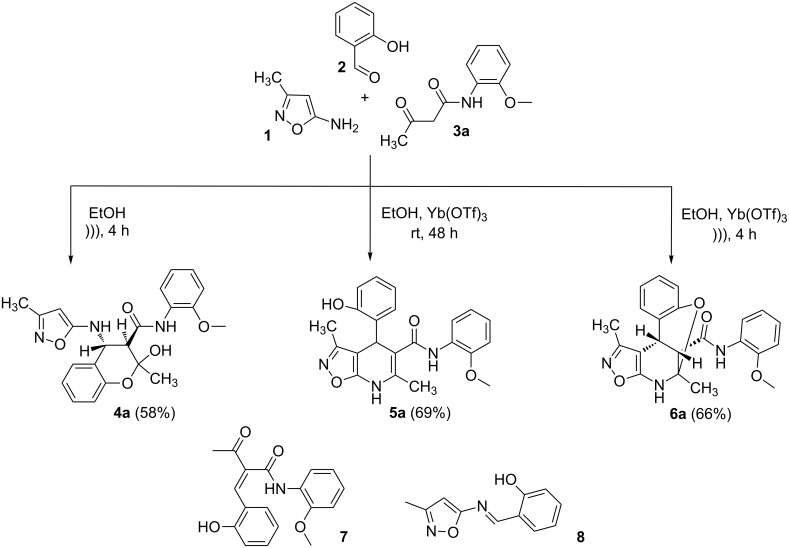
Three-component heterocyclization of 5-amino-3-methylisoxazole (**1**), salicylaldehyde (**2**) and *N*-(2-methoxyphenyl)-3-oxobutanamide (**3a**).

The three-component heterocyclization of 5-amino-3-methylisoxazole (**1**), salicylaldehyde (**2**) and *N*-(2-methoxyphenyl)-3-oxobutanamide (**3a**) was chosen as a model reaction which was studied by variation of the reaction conditions. First, at room temperature under mechanical stirring for 48 h the reaction proceeded to form mainly two compounds – Knoevenagel adduct **7** and Schiff base **8** (Scheme 3). Trace amounts of 2-hydroxy-*N*-(2-methoxyphenyl)-2-methyl-4-(3-methylisoxazol-5-ylamino)chroman-3-carboxamide (**4a**) were detected in the reaction mixture as well.

Furthermore, both conventional and microwave heating did not produce any positive results. Thus, refluxing of the reactants in water, ethanol, dioxane, or application of microwave irradiation at temperatures up to 140 °C only gave rise to the formation of imine **8** (Scheme 3). The reaction time in these cases reached 3 h. On the other hand, refluxing in high boiling solvents (*n*-butanol, DMF, DMSO), as well as microwave irradiation at temperatures above 140 °C, resulted in resinification of the reaction mixture. The reaction times were varied from a minute (for microwave irradiation) to 40 min (for conventional heating).

Inter alia, ultrasonic activation was applied to promote this multicomponent reaction. It was established that the three-component cyclocondensation of the starting compounds under ultrasonication at room temperature for 4 h led to the selective formation of the substituted chroman-3-carboxamide **4a** in 58% yield (Scheme 3). In contrast to the similar reaction involving derivatives of 1,3-cyclohexanedione [13], here the final product was formed with participation of the exocyclic NH_2_-group of aminoisoxazole but not with its 4-CH center that may be linked via different mechanisms (see below).

Next the effect of different catalysts on the reaction was studied. It should be noted that in the case of catalytic processes both conventional and microwave heating were also inefficacious. The only noteworthy result was the detection of trace amounts of 4-(2-hydroxyphenyl)-*N*-(2-methoxyphenyl)-3,6-dimethyl-4,7-dihydroisoxazolo[5,4-*b*]pyridine-5-carboxamide (**5a**) (Scheme 3) in the reaction mixture when the reaction was performed in *n*-butanol under conventional heating for 25 min using ytterbium or scandium triflate as the catalysts.

Then, it was established that the three-component heterocyclization of aminoisoxazole **1**, salicylaldehyde (**2**) and acetoacetamide **3a** in the presence of 5 mol % ytterbium triflate as a catalyst in ethanol under stirring at room temperature for 48 h led to the formation of the aforementioned dihydroisoxazolopyridine **5a** in 69% yield.

To our surprise the analogous catalytic reaction in ethyl alcohol under ultrasonication at room temperature for 4 h gave exclusively *N*-(2-methoxyphenyl)-1,5-dimethyl-5,11-dihydro-4*H*-5,11-methanobenzo[*g*]isoxazolo[5,4-*d*][1,3]oxazocine-12-carboxamide (**6a**) in 66% yield (Scheme 3), while the absence of the isomeric heterocyclic compound **5a** was proven by means of TLC and NMR spectroscopy. In all cases of three-component reactions involving aminoazoles and carbonyl compounds, which were previously studied in our group [13,18,21–24], the substitution of mechanical stirring by ultrasonication did not change the structure of the final products but only affected the purity and yields of the final compounds as well as the reaction rate.

Several Lewis and Brønsted acids have been scanned as catalysts for this reaction. The results of the catalyst system selection for this reaction under stirring at room temperature are summarized in Table 1 and Table 2. Ytterbium triflate (5 mol %) was identified as the catalyst of choice.

**Table 1:** Optimization of the reaction conditions for obtaining compound **5a**.

Catalyst	Catalyst amount (% of the stoichiometric)	Product yield (%)

Sc(OTf)_3_	2	33
Sc(OTf)_3_	5	58
Sc(OTf)_3_	10	54
Sc(OTf)_3_	15	47
Yb(OTf)_3_	2	30
**Yb(OTf)_3_**	**5**	**69**
Yb(OTf)_3_	10	64
Yb(OTf)_3_	15	52
Al(O-iPr)_3_	10	15
Al(O-iPr)_3_	20	18
HCl	5	no product (mixture resinification)
*p*-TsOH	2	no product (mixture resinification)

**Table 2:** Optimization of the reaction conditions for heterocycle **6a** synthesis.

Catalyst	Catalyst amount (% of the stoichiometric)	Product yield (%)

Sc(OTf)_3_	2	27
Sc(OTf)_3_	5	62
Sc(OTf)_3_	10	58
Sc(OTf)_3_	15	38
Yb(OTf)_3_	2	35
**Yb(OTf)_3_**	**5**	**66**
Yb(OTf)_3_	10	59
Yb(OTf)_3_	15	42
Al(O-iPr)_3_	10	no product
Al(O-iPr)_3_	20	15
HCl	5	no product (mixture resinification)
*p*-TsOH	2	no product (mixture resinification)

The next step of our study was to expand the range of *N*-aryl-3-oxobutanamides. First, it is worth mentioning that the three-component cyclocondensation of 5-amino-3-methylisoxazole (**1**), salicylaldehyde (**2**) and *N*-aryl-3-oxobutanamides **3a**–**h** under ultrasonication at room temperature for 4 h without catalyst always led to the selective formation of the corresponding chroman-3-carboxamides **4a**–**h** (Table 3, entries 1, 4, 7, 10, 13, 16, 19, 22).

**Table 3:** Three-component heterocyclization of 5-amino-3-methylisoxazole (**1**), salicylaldehyde (**2**) and *N*-aryl-3-oxobutanamides (**3a**–**h**).

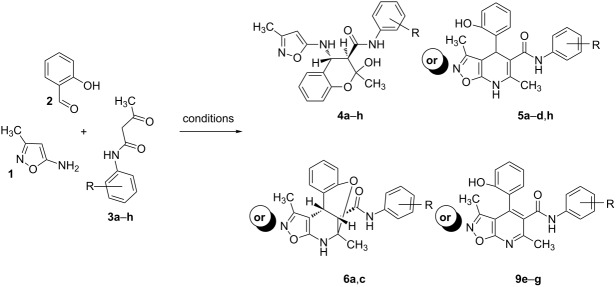				

Entry	Amide	Conditions	Product	Yield, %

1	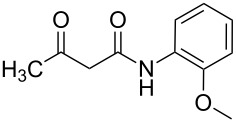 **3a**	EtOH, ))), rt, 4 h	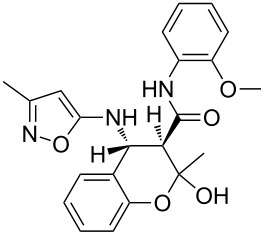 **4a**	58
2		EtOH, Yb(OTf)_3_ (5 mol %), rt, 48 h	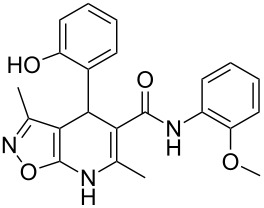 **5a**	69
3		EtOH, Yb(OTf)_3_ (5 mol %), ))), rt, 4 h	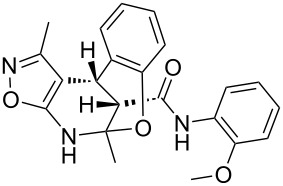 **6a**	66
4	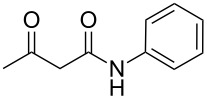 **3b**	EtOH, ))), rt, 4 h	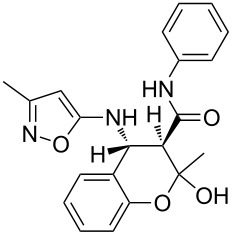 **4b**	54
5		EtOH, Yb(OTf)_3_ (5 mol %), rt, 48 h	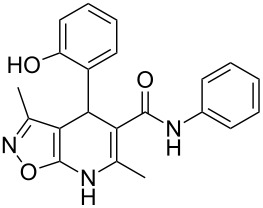 **5b**	70
6		EtOH, Yb(OTf)_3_ (5 mol %), ))), rt, 4 h		69
7	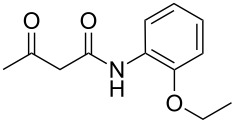 **3c**	EtOH, ))), rt, 4 h	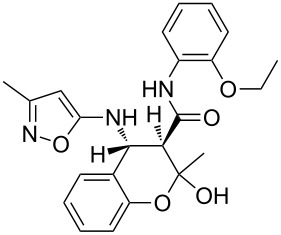 **4c**	51
8		EtOH, Yb(OTf)_3_ (5 mol %), rt, 48 h	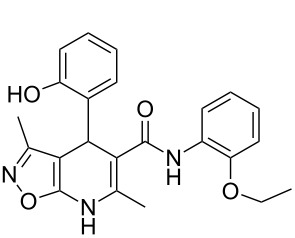 **5c**	68
9		EtOH, Yb(OTf)_3_ (5 mol %), ))), rt, 4 h	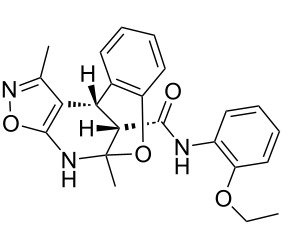 **6c**	64
10	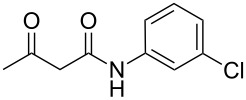 **3d**	EtOH, ))), rt, 4 h	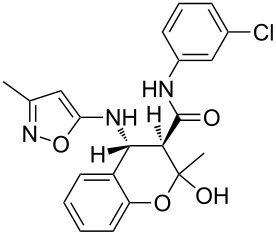 **4d**	58
11		EtOH, Yb(OTf)_3_ (5 mol %), rt, 48 h	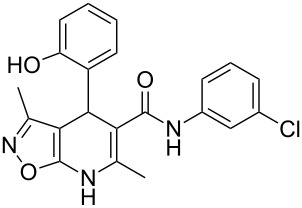 **5d**	61
12		EtOH, Yb(OTf)_3_ (5 mol %), ))), rt, 4 h		65
13	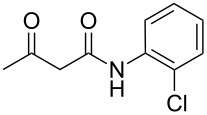 **3e**	EtOH, ))), rt, 4 h	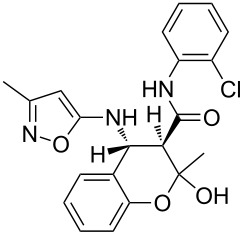 **4e**	57
14		EtOH, Yb(OTf)_3_ (5 mol %), rt, 48 h	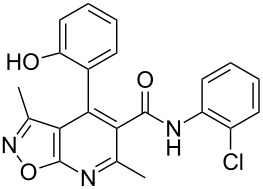 **9e**	72
15		EtOH, Yb(OTf)_3_ (5 mol %), ))), rt, 4 h		81
16	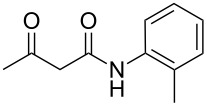 **3f**	EtOH, ))), rt, 4 h	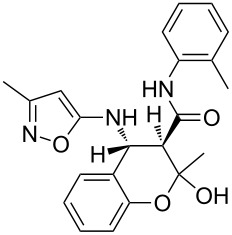 **4f**	59
17		EtOH, Yb(OTf)_3_ (5 mol %), rt, 48 h	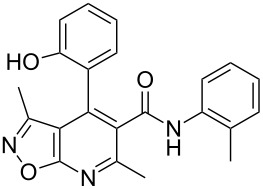 **9f**	77
18		EtOH, Yb(OTf)_3_ (5 mol %), ))), rt, 4 h		75
19	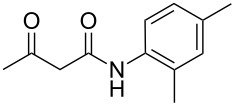 **3g**	EtOH, ))), rt, 4 h	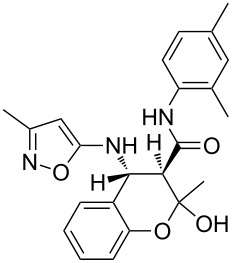 **4g**	63
20		EtOH, Yb(OTf)_3_ (5 mol %), rt, 48 h	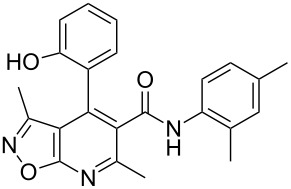 **9g**	76
21		EtOH, Yb(OTf)_3_ (5 mol %), ))), rt, 4 h		82
22	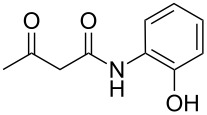 **3h**	EtOH, ))), rt, 4 h	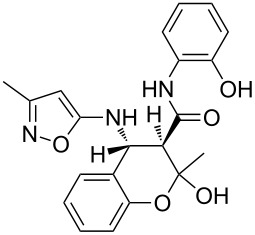 **4h**	55
23		EtOH, Yb(OTf)_3_ (5 mol %), rt, 48 h	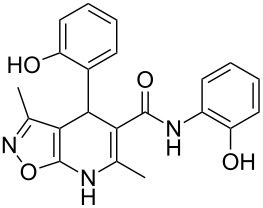 **5h**	61
24		EtOH, Yb(OTf)_3_ (5 mol %), ))), rt, 4 h		63

However, to our surprise, the ytterbium triflate-catalyzed three-component reaction of 5-amino-3-methylisoxazole (**1**), salicylaldehyde (**2**) and *N*-phenyl-3-oxobutanamide (**3b**) gave rise to the formation of 4-(2-hydroxyphenyl)-3,6-dimethyl-*N*-phenyl-4,7-dihydroisoxazolo[5,4-*b*]pyridine-5-carboxamide (**5b**) both under stirring and under ultrasonication at room temperature (Table 3, entries 5, 6). The scope and limitations for this process was therefore studied.

It was shown that *N*-(2-ethoxyphenyl)-3-oxobutanamide (**3c**) behaved under all reaction conditions in the same way as *N*-(2-methoxyphenyl)-3-oxobutanamide (**3a**): the three-component heterocyclization in the presence of ytterbium triflate under stirring at room temperature led to the formation of dihydroisoxazolopyridine **5c** (Table 3, entry 8) whereas the analogous reaction under ultrasonication gave exclusively the benzoxazocine derivative **6c** (Table 3, entry 9).

Interestingly, such a product dichotomy in condensations under or without ultrasonication was not typical for the other studied *N*-aryl-3-oxobutanamides **3b**,**d**–**h**. By this way the 4,7-dihydroisoxazolo[5,4-*b*]pyridine-5-carboxamides **5b**,**d**,**h** were obtained if the corresponding *N*-aryl-3-oxobutanamides contained either an *ortho*-hydroxy group in the benzene ring or no *ortho*-substituent (Table 3, entries 5, 6, 11, 12, 23, 24). On the other hand, *N*-aryl-4-(2-hydroxyphenyl)-3,6-dimethylisoxazolo[5,4-*b*]pyridine-5-carboxamides **9e**–**g** were allocated if the acetoacetamide had any non oxygen-containing *ortho*-substituent on the benzene ring (Table 3, entries 14, 15, 17, 18, 20, 21). It is worth mentioning that aromatic compounds **9e**–**g** were isolated in both aerobic and anaerobic (under inert argon atmosphere) conditions.

**Figure 1:**
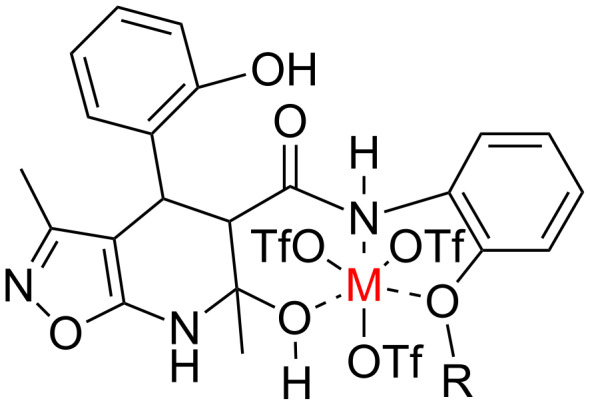
The possible structure of an intermediate complex in reactions forming the heterocycles **6**.

Based on these investigations we have proposed the structure of a feasible intermediate complex in the catalytic reactions leading to compounds **6** (Figure 1). This complex presumably facilitates a subsequent nucleophilic substitution of the salicylic hydroxy group leading to the oxygen-bridged heterocycle **6**. Apparently the oxygen-containing *ortho*-substituent on the benzene ring adopts an important role in the central ion coordination.

At first glance, the behavior of *N*-(2-hydroxyphenyl)-3-oxobutanamide (**3h**) in the three-component catalytic reaction with 5-amino-3-methylisoxazole (**1**) and salicylaldehyde (**2**) does not confirm this hypothesis since compound **6** was not observed in this case (Table 3, entries 23, 24). As one possible reason we assumed that the presence of an acidic phenol group – distinguishing the amide **3h** from alkoxylated amides **3a**,**c** – could be responsible for a destabilization of the aforementioned complex. Indeed, it was established that the three-component heterocyclizations of aminoisoxazole **1** with salicylaldehyde (**2**) and *N*-aryl-3-oxobutanamides **3a**,**c** supplemented with ytterbium triflate as a catalyst in the presence of an equimolar amount of phenol (as a source of phenolic acidity) led to the formation of dihydroisoxazolopyridines **5a**,**c** rather than benzoxazocines **6a,c** thus supporting the assumptions.

**Scheme 4:**
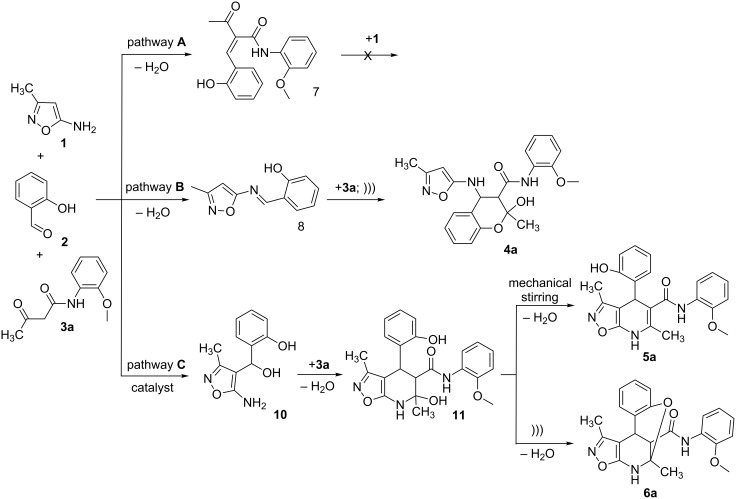
Possible pathways for the three-component reaction of 5-amino-3-methylisoxazole (**1**), salicylaldehyde (**2**) and *N*-(2-methoxyphenyl)-3-oxobutanamide (**3a**).

A plausible mechanistic rationale is outlined in Scheme 4. It should be firstly noted that the formation of any of the compounds **4**–**6a** through the intermediate **7** (pathway A) as for cyclic 1,3-diketones [13,25] was excluded because the latter does not react with amide **3a** under any comparable conditions.

Most likely the formation of chroman-3-carboxamide **4a** proceeds through imine **8** (pathway B) since the reaction of **8** and **3a** under ultrasonication at room temperature gave the corresponding final product essentially after the same reaction time.

The Lewis acid catalyst activates the carbonyl group in salicylaldehyde (**2**), which is subsequently able to interact with the 4-CH nucleophilic center of the aminoisoxazole **1** and not only with the exocyclic NH_2_ group [26]. The resulting adduct **10** (Scheme 4, pathway C) then reacts with carboxamide **3a** forming intermediate **11** which can lose a water molecule in two different ways: either by elimination occurring at room temperature leading to dihydroisoxazolo[5,4-*b*]pyridine **5a** or through nucleophilic substitution involving the phenolic hydroxy group under ultrasonic activation furnishing the oxygen-bridged compound **6a**. Most likely, here the key influence of ultrasound is the transfer of energy to the reaction mixture that is required for intramolecular cyclization that cannot be received by standard mechanical stirring.

However, it cannot be excluded that the Lewis acid catalyzed process proceeds through a primary attack of the exocyclic NH_2_ group of aminoisoxazole to the activated β-carbonyl group of acetoacetamide and further reaction with the aldehyde, as it was described for analogous three-component reactions [27–28].

The structures of all synthesized compounds were unambiguously established by elemental analysis, MS, NMR spectroscopy and X-ray analysis. Thus, the ^1^H NMR spectrum of chroman-3-carboxamide **4a** exhibits a singlet for the NH amide group at δ 9.25, a broad multiplet including peaks for the aromatic protons, a singlet for the OH group, and a doublet for the NH-isoxazole group around δ 6.70–7.84, a multiplet for the 4-CH-group at δ 4.90, a singlet for the CH-isoxazole group at δ 4.71, a doublet for the 3-CH-group at δ 2.94 as well as signals for the other terminal substituents. The relative stereochemistry of the stereogenic centers at positions 3 and 4 of compound **4a** was established by 1D and 2D NMR spectra. Thus, a *^3^J* coupling constant of 11.7 Hz accounts for a *trans*-orientation. The NOESY experiment showed only a quite weak interaction between these protons.

**Figure 2:**
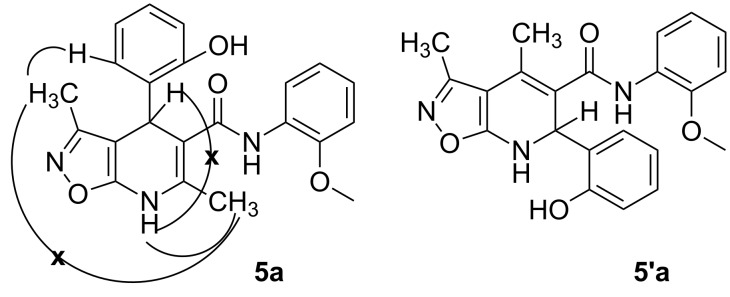
Alternative structures **5a** and **5'a** for dihydroisoxazolopyridine **5a** and selected NOESY correlations.

The spectral data obtained for dihydroisoxazolopyridine **5a** may correspond to at least two possible isomers **5a** and **5'a** (Figure 2). The data from COSY and NOESY experiments showed correlations between protons of NH and CH_3_ groups in the dihydropyridine ring and no interactions between NH and CH groups or two CH_3_ groups supporting structure **5'a** could be detected. All the data obtained from HMBC spectra also unambiguously support the connectivity of structure **5a** but not of isomer **5'a**.

**Figure 3:**
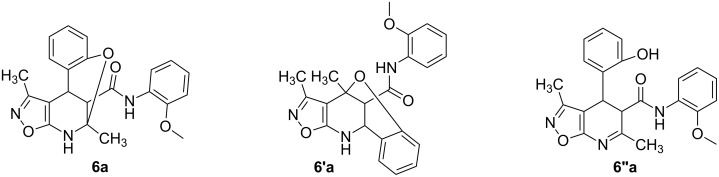
Alternative structures **6a**, **6'a** and **6''a** for compound **6a**.

**Figure 4:**
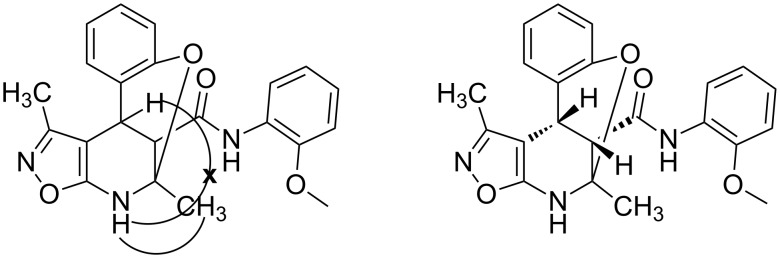
Selected data from NOESY experiments and relative stereochemistry of stereogenic centers at positions 4 and 12.

The spectra observed for compound **6a** may correspond to at least three possible isomers **6a**, **6'a**, and **6''a** (Figure 3). Assignment of the structure **6a** was achieved with the help of 2D NMR experiments (Figure 4).

COSY and NOESY spectra showed correlations between the protons of the NH and CH_3_ groups in the tetrahydropyridine ring which should be absent in case of structure **6''a**. Neither scalar nor dipolar coupling between NH and CH groups were detected in these experiments which would be expected for structure **6'a**. The data obtained from the HMBC spectrum also unambiguously supported the connectivity of structure **6a** and not those of structures **6'a** or **6''a**.

The relative stereochemistry of the stereogenic centers at positions 4 and 12 of compound **6a** was first established by analysis of the 1D and 2D NMR spectra. Thus, a *^3^J* coupling constant of 1.9 Hz corresponds with a *cis*-orientation and the NOESY experiment additionally supports the proximity of this pair of protons.

**Figure 5:**
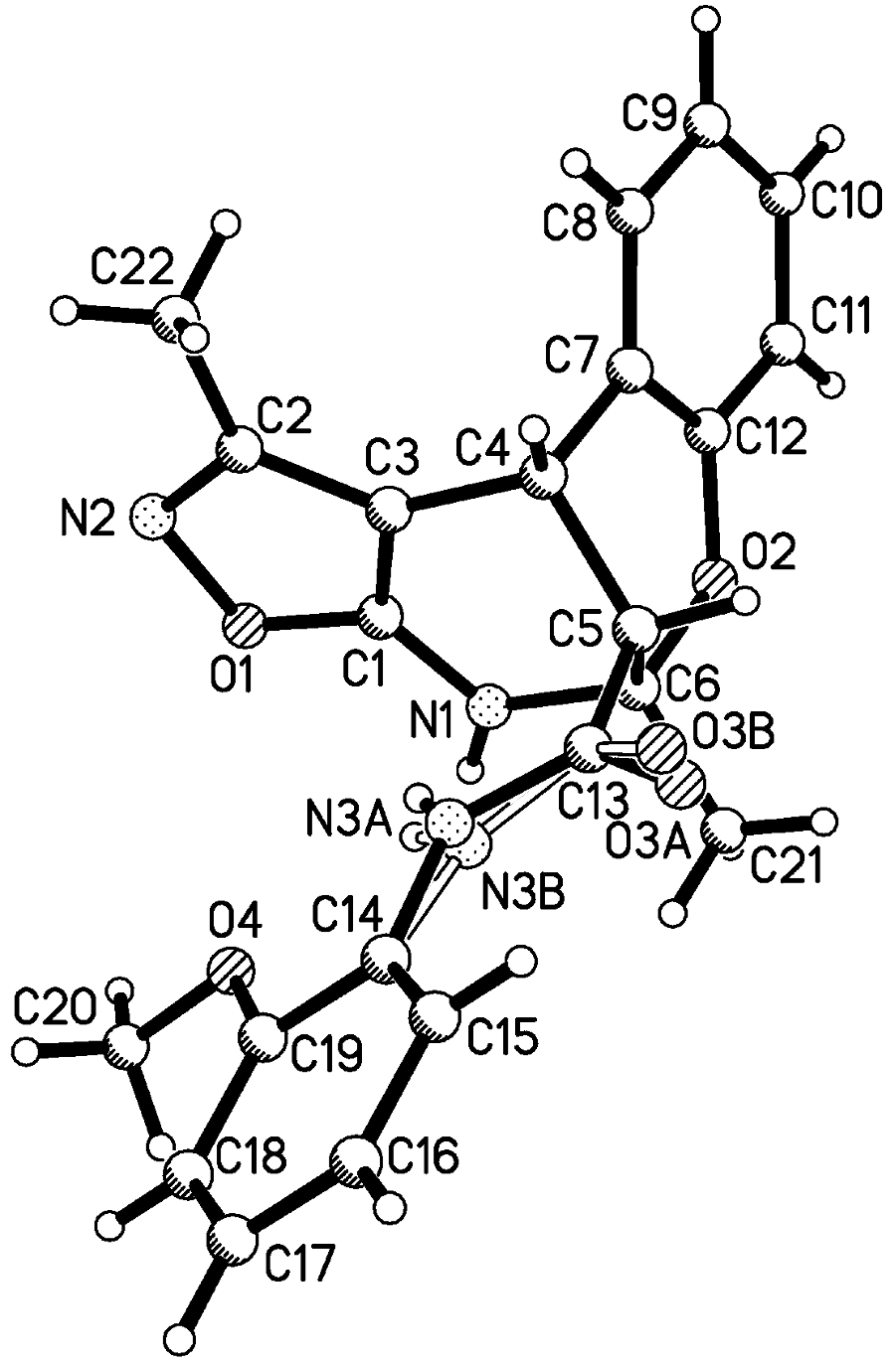
Molecular structure of compound **6a** according to X-ray diffraction data.

The structure of compound **6a** with relative stereochemistry of stereogenic centers was finally corroborated by an X-ray diffraction study (Figure 5). The heterocycles of the polycyclic fragment adopt a chair-like conformation (the puckering parameters [29] are: S = 0.78, Θ = 32.8°, Ψ = 1.0° and S = 0.83, Θ = 38.0°, Ψ = 1.8° for the tetrahydropyridine and dihydrooxine rings, respectively). Deviation of the C5 atom from the mean plane of the remaining atoms of the ring is 0.70 Å and −0.77 Å for these two rings.

## Conclusion

In summary, the three-component heterocyclizations involving 5-amino-3-methylisoxazole, salicylaldehyde and *N*-aryl-3-oxobutanamides were studied in detail. Generally the reaction may be switched between two pathways leading to the formation of 4-(isoxazol-5-ylamino)chroman-3-carboxamides (ultrasonication at room temperature without catalysts) and isoxazolo[5,4-*b*]pyrimidine-5-carboxamides (ultrasonication or mechanical stirring at room temperature with Yb(OTf)_3_). However, an unexpected influence of the *N*-*o*-alkoxyaryl substituent in acetoacetamides on the outcome of the process under ultrasonication leading to the exclusive isolation of benzo[*g*]isoxazolo[5,4-*d*][1,3]oxazocine-12-carboxamides was additionally observed. The mechanistic rationale was developed on the basis of experiments with presumed intermediates and literature evidence.

## Supporting Information

File 1 Experimental procedures, characterization data, ^1^H and ^13^C spectra for all new compounds.
