# Supplementary material for: The unexpected influence of aryl substituents in N-aryl-3-oxobutanamides on the behavior of their multicomponent reactions with 5-amino-3-methylisoxazole and salicylaldehyde
Source: Beilstein J Org Chem. 2014 Dec 17;10:3019–30. doi: 10.3762/bjoc.10.320 (PMC4311697; doi:10.3762/bjoc.10.320)
Supplement: File 1 — Experimental procedures, characterization data, 1H and 13C spectra for all new compounds. [file Beilstein_J_Org_Chem-10-3019-s001.pdf]

Supporting Information
for
The unexpected influence of aryl substituents in *N*-aryl-3-oxobutanamides on the behavior of their multicomponent reactions with 5-amino-3-methylisoxazole and salicylaldehyde

Volodymyr V. Tkachenko[1,2], Elena A. Muravyova[1], Sergey M. Desenko[1,2], Oleg v. Shishkin[1,2], Svetlana V. Shishkina[1], Dmytro O. Sysoiev[3], Thomas J. J. Müller[4], and Valentin A. Chebanov*[1,2,4]

Address: [1]Division of Chemistry of Functional Materials, SSI “Institute for Single Crystals” NAS of Ukraine, Lenin Ave. 60, Kharkiv 61001, Ukraine, [2]Faculty of Chemistry, V. N. Karazin Kharkiv National University, Svobody sq. 4, Kharkiv 61022, Ukraine, [3]Fachbereich Chemie, University of Konstanz, Fach M-720, Universitaetsstrasse 10, Konstanz 78457, Germany and [4]Institut für Organische Chemie und Makromolekulare Chemie, Heinrich-Heine-Universität Düsseldorf, Universitätsstraße 1, Düsseldorf D-40225, Germany

Email: Valentin A. Chebanov - chebanov@isc.kharkov.com

*Corresponding author

Experimental procedures, characterization data, ^{1}H and ^{13}C spectra for all new compounds

Experimental part

Solvents and all reagents were obtained from standard commercial vendors and used without additional purification.

Sonication was carried out with help of standard ultrasonic bath producing irradiation at 44.2 kHz in a round-bottomed flask equipped with a condenser.

Melting points were measured on a standard melting point apparatus in open capillary tubes and were uncorrected. The ^{1}H NMR spectra were recorded in DMSO-d$_6$ at 600 MHz (150 MHz for ^{13}C) for 2D NMR and at 200 MHz (50 MHz for ^{13}C) for 1D NMR on Bruker Avance DRX600 and Varian Mercury VX-200 spectrometers. High resolution mass spectra were measured on a GC-MS Varian 1200L (ionizing voltage 70 eV, direct input of the sample). Elemental analysis was made on a EuroVector EA-3000. TLC analyses were carried out on Merck plates coated with silica gel (60 F 254).

General procedure for the preparation of *N*-aryl-2-hydroxy-2-methyl-4-(3-methylisoxazol-5-ylamino)-3,4-dihydro-2*H*-chromene-3-carboxamides 4a-h. The mixture containing 1 mmol of 3-methylisoxazol-5-amine (**1**), 1 mmol of salicylaldehyde (**2**), 1 mmol of the appropriate *N*-aryl-3-oxobutanamide **3a-h** and 3 mL of ethanol was continuously ultrasonicated at room temperature for 4 h. Then the reaction mixture was set aside for a day. The solid precipitated was collected by filtration and vacuum dried. The target compounds were obtained in high purity (ca. 95% according to TLC and NMR and elemental analysis) and no additional recrystallization was needed. Some of the compounds **4** were isolated as a mixture of diastereomers. In these cases only the signals of major diastereomer are given for NMR spectra. Also, these compounds become unstable even with a slight heating and are prone to decay in NMR tube.

2-Hydroxy-*N*-(2-methoxyphenyl)-2-methyl-4-(3-methylisoxazol-5-ylamino)-3,4-dihydro-2*H*-chromene-3-carboxamide (4a). Yield 58%, colorless solid, m.p. 173–174 °C. ^{1}H NMR (DMSO-*d$_6$*) δ 9.35 (s, 1H, NH), 7.84–6.70 (m, 10H, ArH + OH + NH), 4.90 (m, 1H, CH), 4.71 (s, 1H, CH), 3.80 (s, 3H, CH_3O), 2.94 (d, J = 11.7 Hz, 1H, CH), 1.92 (s, 3H, CH_3), 1.62 (s, 3H, CH_3) ppm. ^{13}C NMR (DMSO-*d$_6$*) δ 170.23, 168.19, 160.54, 152.00, 149.53, 129.05, 127.76, 127.08, 124.58, 124.38, 121.27, 121.22, 120.80, 120.69, 117.14, 111.52, 97.60, 77.76, 56.34, 50.93, 26.74, 11.64 ppm. MS (EI, 70 eV): *m/z* (%) = 409 (13) [M$^+$], 391 (48), 123

(100), 43 (49). Anal. Calcd. for $C_{22}H_{23}N_3O_5$: C 64.54, H 5.66, N 10.26. Found: C 64.32, H 5.90, N 10.41%.

2-Hydroxy-2-methyl-4-(3-methylisoxazol-5-ylamino)-*N*-phenyl-3,4-dihydro-2*H*-chromene-3-carboxamide (4b). Yield 54%, colorless solid, m.p. 185–186 °C. ^{1}H NMR (DMSO-d_6) δ 9.92 (s, 1H, NH), 7.67–6.79 (m, 11H, ArH + OH + NH), 5.14 (m, 1H, CH), 4.89 (s, 1H, CH), 3.05 (d, J = 11.2 Hz, 1H, CH), 2.00 (s, 3H, CH_3), 1.64 (s, 3H, CH_3) ppm. ^{13}C NMR (DMSO-d_6) δ 170.06, 167.39, 160.08, 151.55, 138.81, 128.71, 128.65, 128.51, 125.60, 124.40, 123.39, 120.65, 119.42, 119.14, 116.60, 97.56, 77.36, 54.09, 49.28, 26.64, 11.29 ppm. MS (EI, 70 eV): *m/z* (%) = 379 (13) [M^+], 361 (45), 177 (36), 93 (100). Anal. Calcd. for $C_{21}H_{21}N_3O_4$: C 66.48, H 5.58, N 11.08. Found: C 66.59, H 5.44, N 11.00%.

***N*-(2-Ethoxyphenyl)-2-hydroxy-2-methyl-4-(3-methylisoxazol-5-ylamino)-3,4-dihydro-2*H*-chromene-3-carboxamide (4c).** Yield 51%, colorless solid, m.p. 182–183 °C. ^{1}H NMR (DMSO-d_6) δ 9.38 (s, 1H, NH), 7.81–6.74 (m, 10H, ArH + OH + NH), 5.01 (m, 1H, CH), 4.81 (s, 1H, CH), 4.08 (q, J = 7.0 Hz, 2H, $CH_3\underline{CH_2}O$), 3.03 (d, J = 11.8 Hz, 1H, CH), 1.94 (s, 3H, CH_3), 1.63 (s, 3H, CH_3), 1.36 (t, J = 7.0 Hz, 3H, $\underline{CH_3}CH_2O$) ppm. ^{13}C NMR (DMSO-d_6) δ 175.40, 162.80, 159.39, 154.09, 148.85, 130.85, 129.72, 127.59, 125.84, 122.96, 122.60, 122.32, 120.87, 118.47, 114.54, 93.32, 86.68, 64.51, 58.48, 44.52, 25.48, 13.82, 11.08 ppm. MS (EI, 70 eV): *m/z* (%) = 423 (17) [M^+], 405 (53), 137 (100), 54 (36). Anal. Calcd. for $C_{23}H_{25}N_3O_5$: C 65.24, H 5.95, N 9.92. Found: C 65.22, H 6.00, N 9.95%.

***N*-(3-Chlorophenyl)-2-hydroxy-2-methyl-4-(3-methylisoxazol-5-ylamino)-3,4-dihydro-2*H*-chromene-3-carboxamide (4d).** Yield 58%, colorless solid, m.p. 170–171 °C. ^{1}H NMR (DMSO-d_6) δ 10.12 (s, 1H, NH), 7.82–6.78 (m, 10H, ArH + OH + NH), 5.11 (m, 1H, CH), 4.87 (s, 1H, CH), 3.00 (d, J = 11.0 Hz, 1H, CH), 1.98 (s, 3H, CH_3), 1.59 (s, 3H, CH_3) ppm. ^{13}C NMR (DMSO-d_6) δ 170.53, 168.31, 160.63, 152.00, 140.68, 133.53, 130.91, 128.90, 127.13, 124.77, 123.64, 121.21, 119.38, 118.25, 117.14, 97.94, 77.91, 54.81, 49.76, 27.14, 11.79 ppm. MS (EI, 70 eV): *m/z* (%) = 415 (5), 413 (16) [M^+], 397 (14), 395 (40), 129 (36), 127 (100), 43 (20). Anal. Calcd. for $C_{21}H_{20}ClN_3O_4$: C 60.95, H 4.87, N 10.15. Found: C 61.12, H 4.69, N 10.01%.

***N*-(2-Chlorophenyl)-2-hydroxy-2-methyl-4-(3-methylisoxazol-5-ylamino)-3,4-dihydro-2*H*-chromene-3-carboxamide (4e).** Yield 57%, colorless solid, m.p. 190–191 °C. ^{1}H NMR (DMSO-d_6) δ 10.01 (s, 1H, NH), 7.81–6.91 (m, 10H, ArH + OH + NH), 5.02 (m, 1H, CH), 4.83 (s, 1H, CH), 3.05 (d, J = 11.8 Hz, 1H, CH), 1.94 (s, 3H, CH_3), 1.62 (s, 3H, CH_3) ppm.

^{13}C NMR (DMSO-d_6) δ 170.27, 168.48, 160.58, 151.99, 135.17, 129.82, 129.10, 127.94, 127.16, 126.44, 125.96, 125.35, 124.32, 121.31, 117.19, 97.60, 77.92, 55.33, 50.73, 26.95, 11.76 ppm. MS (EI, 70 eV): *m/z* (%) = 415 (4), 413 (13) [M^+], 397 (17), 395 (53), 129 (34), 127 (100). Anal. Calcd. for $C_{21}H_{20}ClN_3O_4$: C 60.95, H 4.87, N 10.15. Found: C 60.99, H 4.91, N 10.19%.

2-Hydroxy-2-methyl-4-(3-methylisoxazol-5-ylamino)-*N*-(*o*-tolyl)-3,4-dihydro-2*H*-chromene-3-carboxamide (4f). Yield 59%, colorless solid, m.p. 178–179 °C. ^1H NMR (DMSO-d_6) δ 9.33 (s, 1H, NH), 7.66–6.76 (m, 10H, ArH + OH + NH), 5.06 (m, 1H, CH), 4.87 (s, 1H, CH), 3.03 (d, J = 12.7 Hz, 1H, CH), 2.12 (s, 3H, CH_3), 1.97 (s, 3H, CH_3), 1.67 (s, 3H, CH_3) ppm. ^{13}C NMR (DMSO-d_6) δ 170.45, 168.12, 160.52, 152.08, 136.63, 132.29, 130.76, 130.72, 128.93, 127.16, 126.36, 125.74, 125.44, 124.74, 121.17, 117.12, 97.87, 77.83, 50.37, 27.17, 18.15, 11.79 ppm. MS (EI, 70 eV): *m/z* (%) = 393 (9) [M^+], 375 (24), 107 (100), 43 (35). Anal. Calcd. for $C_{22}H_{23}N_3O_4$: C 67.16, H 5.89, N 10.68. Found: C 67.05, H 5.77, N 10.55%.

***N*-(2,4-Dimethylphenyl)-2-hydroxy-2-methyl-4-(3-methylisoxazol-5-ylamino)-3,4-dihydro-2*H*-chromene-3-carboxamide (4g).** Yield 63%, colorless solid, m.p. 183–184 °C. ^1H NMR (DMSO-d_6) δ 9.26 (s, 1H, NH), 7.65–6.76 (m, 9H, ArH + OH + NH), 5.02 (m, 1H, CH), 4.86 (s, 1H, CH), 3.01 (d, J = 12.0 Hz, 1H, CH), 2.21 (s, 3H, CH_3), 2.06 (s, 3H, CH_3), 1.97 (s, 3H, CH_3), 1.66 (s, 3H, CH_3) ppm. ^{13}C NMR (DMSO-d_6) δ 170.45, 168.10, 160.50, 152.09, 134.85, 134.05, 132.35, 131.22, 128.90, 127.16, 126.83, 125.57, 124.77, 121.14, 117.10, 97.90, 77.80, 54.54, 50.33, 27.18, 20.94, 18.08, 11.80 ppm. MS (EI, 70 eV): *m/z* (%) = 407 (17) [M^+], 389 (66), 121 (100), 43 (43). Anal. Calcd. for $C_{23}H_{25}N_3O_4$: C 67.80, H 6.18, N 10.31. Found: C 67.94, H 6.08, N 10.23%.

2-Hydroxy-*N*-(2-hydroxyphenyl)-2-methyl-4-(3-methylisoxazol-5-ylamino)-3,4-dihydro-2*H*-chromene-3-carboxamide (4h). Yield 55%, colorless solid, m.p. 195–196 °C. ^1H NMR (DMSO-d_6) δ 9.86 (s, 1H, NH), 9.39 (s, 1H, OH), 7.83–6.73 (m, 10H, ArH + OH + NH), 4.98 (m, 1H, CH), 4.79 (s, 1H, CH), 3.02 (d, J = 11.9 Hz, 1H, CH), 1.92 (s, 3H, CH_3), 1.62 (s, 3H, CH_3) ppm. ^{13}C NMR (DMSO-d_6) δ 175.48, 162.52, 159.25, 154.68, 149.51, 129.32, 128.12, 127.20, 126.43, 123.91, 122.16, 120.97, 120.84, 117.99, 115.96, 93.72, 86.09, 58.11, 44.80, 25.40, 11.09 ppm. MS (EI, 70 eV): *m/z* (%) = 395 (15) [M^+], 377 (53), 109 (100), 54 (49). Anal. Calcd. for $C_{21}H_{21}N_3O_5$: C 63.79, H 5.35, N 10.63. Found: C 63.60, H 5.12, N 10.47%.

General procedure for the preparation of *N*-aryl-4-(2-hydroxyphenyl)-3,6-dimethyl-4,7-dihydroisoxazolo[5,4-*b*]pyridine-5-carboxamides 5a-d,h. The mixture containing 1 mmol of 3-methylisoxazol-5-amine (**1**), 1 mmol of salicylaldehyde (**2**), 1 mmol of the appropriate *N*-aryl-3-oxobutanamide **3a-d,h**, 0.05 mmol of ytterbium triflate and 3 mL of ethanol was continuously stirred at room temperature for 48 h. The solid precipitated was collected by filtration and vacuum dried. The target compound was obtained in high purity (ca. 95% according to TLC and NMR) and no additional recrystallization was needed.

4-(2-Hydroxyphenyl)-*N*-(2-methoxyphenyl)-3,6-dimethyl-4,7-dihydroisoxazolo[5,4-*b*]pyridine-5-carboxamide (5a). Yield 69%, colorless solid, m.p. 198–199 °C. ^{1}H NMR (DMSO-d_6) δ 9.89 (s, 1H, NH), 9.62 (s, 1H, OH), 8.12 (s, 1H, NH), 7.90–6.76 (m, 8H, ArH), 5.37 (s, 1H, CH), 3.67 (s, 3H, CH_3O), 2.21 (s, 3H, CH_3), 1.90 (s, 3H, CH_3) ppm. ^{13}C NMR (DMSO-d_6) δ 166.28, 160.03, 157.78, 153.19, 148.75, 139.78, 131.39, 129.75, 127.64, 127.58, 123.54, 120.59, 120.08, 119.70, 115.05, 110.66, 105.83, 93.75, 55.99, 55.47, 18.52, 9.75 ppm. MS (EI, 70 eV): *m/z* (%) = 391 (34) [M^+], 374 (14), 123 (100), 108 (19). Anal. Calcd. for $C_{22}H_{21}N_3O_4$: C 67.51, H 5.41, N 10.74. Found: C 67.47, H 5.44, N 10.73%.

4-(2-Hydroxyphenyl)-3,6-dimethyl-*N*-phenyl-4,7-dihydroisoxazolo[5,4-*b*]pyridine-5-carboxamide (5b). Yield 70%, colorless solid, m.p. 204–205 °C. ^{1}H NMR (DMSO-d_6) δ 9.66 (s, 1H, OH), 9.49 (s, 1H, NH), 9.41 (s, 1H, NH), 7.49–6.73 (m, 9H, ArH), 5.54 (s, 1H, CH), 2.08 (s, 3H, CH_3), 1.81 (s, 3H, CH_3) ppm. ^{13}C NMR (DMSO-d_6) δ 166.98, 160.45, 157.84, 153.18, 139.35, 135.99, 131.66, 130.30, 128.69, 128.38 (2C), 127.20, 122.78, 119.35 (2C), 114.72, 107.91, 93.18, 31.99, 17.72, 9.60 ppm. MS (EI, 70 eV): *m/z* (%) = 361 (10) [M^+], 344 (31), 93 (100), 65 (45). Anal. Calcd. for $C_{21}H_{19}N_3O_3$: C 69.79, H 5.30, N 11.63. Found: C 69.90, H 5.23, N 11.69%.

***N*-(2-Ethoxyphenyl)-4-(2-hydroxyphenyl)-3,6-dimethyl-4,7-dihydroisoxazolo[5,4-*b*]pyridine-5-carboxamide (5c).** Yield 68%, colorless solid, m.p. 221–222 °C. ^{1}H NMR (DMSO-d_6) δ 9.82 (s, 1H, NH), 9.53 (s, 1H, OH), 8.20 (s, 1H, NH), 7.76–6.74 (m, 8H, ArH), 5.37 (s, 1H, CH), 3.97 (q, J = 7.0 Hz, 2H, $CH_3C\underline{H_2}O$), 2.17 (s, 3H, CH_3), 1.85 (s, 3H, CH_3), 1.24 (t, J = 7.0 Hz, 3H, $C\underline{H_3}CH_2O$) ppm. ^{13}C NMR (DMSO-d_6) δ 166.91, 158.89, 151.89, 149.57, 147.22, 130.39, 130.15, 130.07, 129.89, 126.28, 122.91, 122.54, 119.90 (2C), 116.51, 114.48, 95.08, 93.27, 55.51, 33.45, 17.50, 13.82, 9.34 ppm. MS (EI, 70 eV): *m/z* (%) = 405 (21) [M^+], 388 (18), 269 (22), 137 (100). Anal. Calcd. for $C_{23}H_{23}N_3O_4$: C 68.13, H 5.72, N 10.36. Found: C 68.07, H 5.67, N 10.34%.

***N*-(3-Chlorophenyl)-4-(2-hydroxyphenyl)-3,6-dimethyl-4,7-dihydroisoxazolo[5,4-*b*]pyridine-5-carboxamide (5d).** Yield 61%, colorless solid, m.p. 228–229 °C. ^{1}H NMR (DMSO-d_6) δ 9.72 (s, 1H, NH), 9.64 (s, 1H, OH), 9.48 (s, 1H, NH), 7.66–6.67 (m, 8H, ArH), 5.52 (s, 1H, CH), 2.04 (s, 3H, CH_3), 1.78 (s, 3H, CH_3) ppm. ^{13}C NMR (DMSO-d_6) δ 167.83, 160.94, 158.36, 153.72, 141.37, 137.02, 133.33, 132.07, 130.76, 130.59, 127.78, 122.92, 119.92, 119.20, 118.13, 115.27, 108.16, 93.67, 32.53, 18.21, 10.10 ppm. MS (EI, 70 eV): *m/z* (%) = 397 (4), 395 (11) [M^+], 269 (100), 129 (9), 127 (29). Anal. Calcd. for $C_{21}H_{18}ClN_3O_3$: C 63.72, H 4.58, N 10.62. Found: C 63.78, H 4.54, N 10.60%.

***N*,4-Bis(2-hydroxyphenyl)-3,6-dimethyl-4,7-dihydroisoxazolo[5,4-*b*]pyridine-5-carboxamide (5h).** Yield 61%, colorless solid, m.p. 216–217 °C. ^{1}H NMR (DMSO-d_6) δ 9.73 (s, 1H, NH), 9.54 (s, 1H, OH), 9.46 (s, 1H, OH), 8.68 (s, 1H, NH), 7.49–6.68 (m, 8H, ArH), 5.46 (s, 1H, CH), 2.14 (s, 3H, CH_3), 1.82 (s, 3H, CH_3) ppm. ^{13}C NMR (DMSO-d_6) δ 167.97, 160.79, 158.35, 153.79, 148.24, 138.30, 131.96, 130.49, 127.95, 127.00, 125.08, 122.38, 120.02, 119.45, 116.56, 115.49, 107.06, 93.85, 32.74, 18.57, 10.15 ppm. MS (EI, 70 eV): *m/z* (%) = 377 (11) [M^+], 360 (14), 269 (21), 109 (100). Anal. Calcd. for $C_{21}H_{19}N_3O_4$: C 66.83, H 5.07, N 11.13. Found: C 66.89, H 5.12, N 11.08%.

General procedure for the preparation of *N*-aryl-1,5-dimethyl-5,11-dihydro-4*H*-5,11-methanobenzo[g]isoxazolo[5,4-*d*][1,3]oxazocine-12-carboxamides 6a,c. The mixture containing 1 mmol of 3-methylisoxazol-5-amine (**1**), 1 mmol of salicylaldehyde (**2**), 1 mmol of the appropriate *N*-aryl-3-oxobutanamide **3a,c**, 0.05 mmol of ytterbium triflate and 3 mL of ethanol was continuously ultrasonicated at room temperature for 4 h. The solid precipitated was collected by filtration and vacuum dried. The target compound was obtained in high purity (ca. 95% according to TLC and NMR) and no additional recrystallization was needed.

***N*-(2-Methoxyphenyl)-1,5-dimethyl-5,11-dihydro-4*H*-5,11-methanobenzo[g]isoxazolo[5,4-*d*][1,3]oxazocine-12-carboxamide (6a).** Yield 66%, colorless solid, m.p. 210–211 °C. ^{1}H NMR (DMSO-d_6) δ 9.35 (s, 1H, NH), 8.80 (s, 1H, NH), 7.87–6.74 (m, 8H, ArH), 4.16 (d, J = 1.9 Hz, 1H, CH), 3.78 (s, 3H, CH_3O), 3.44 (d, J = 1.9 Hz, 1H, CH), 2.07 (s, 3H, CH_3), 1.76 (s, 3H, CH_3) ppm. ^{13}C NMR (DMSO-d_6) δ 167.62, 165.70, 156.69, 152.85, 150.06, 129.22, 127.81, 127.51, 127.12, 125.05, 122.21, 120.71, 120.56, 116.48, 111.63, 92.66, 85.50, 56.17, 45.88, 31.94, 25.01, 10.03 ppm. MS (EI, 70 eV): *m/z* (%) = 391 (25) [M^+], 374 (37), 228 (17), 123 (100), 108 (50). Anal. Calcd. for $C_{22}H_{21}N_3O_4$: C 67.51, H 5.41, N 10.74. Found: C 67.45, H 5.34, N 10.76%.

X-ray crystallographic study. The colourless crystals of **6a** ($C_{22}H_{21}N_3O_4$) are monoclinic. At 293 K a = 12.3582(4), b = 8.9291(2), c = 18.5899(5) Å, β = 105.094(3)°, V = 1980.58(9) Å^3, M_r = 391.42, Z = 4, space group $P2_1/n$, d_{calc} = 1.313 g/cm^3, $\mu(MoK_\alpha)$ = 0.092 mm^{-1}, F(000) = 824. Intensities of 19928 reflections (5778 independent, R_{int} = 0.025) were measured on the "Xcalibur-3" diffractometer (graphite monochromated MoK_α radiation, CCD detector, ω-scanning, $2\Theta_{max}$ = 60°). The structure was solved by direct method using SHELXTL package [1]. The restrains for the bond lengths in the disordered carbamide fragment (over two position with equal population) (C_{sp2}-O 1.21 Å, C_{sp2}-N 1.33 Å, C_{ar}-N 1.35 Å) were applied during refinement. Positions of the hydrogen atoms were located from electron density difference maps and refined by "riding" model with $U_{iso} = nU_{eq}$ (n = 1.5 for methyl groups and n = 1.2 for other hydrogen atoms) of the carrier atom. The hydrogen atoms of the NH groups were refined using isotropic approximation. Full-matrix least-squares refinement against F^2 in anisotropic approximation for non-hydrogen atoms using 5704 reflections was converged to wR_2 = 0.141 (R_1 = 0.049 for 3848 reflections with F > 4σ(F), S = 1.074). The final atomic coordinates and crystallographic data for molecule **6a** have been deposited to with the Cambridge Crystallographic Data Centre, 12 Union Road, CB2 1EZ, UK (fax: +44-1223-336033; e-mail: deposit@ccdc.cam.ac.uk) and are available on request quoting the deposition numbers CCDC 958690.

***N*-(2-Ethoxyphenyl)-1,5-dimethyl-5,11-dihydro-4*H*-5,11-methanobenzo[g]isoxazolo[5,4-*d*][1,3]oxazocine-12-carboxamide (6c).** Yield 64%, colorless solid, m.p. 225–226 °C. ^{1}H NMR (DMSO-*d₆*) *δ* 9.25 (s, 1H, NH), 8.83 (s, 1H, NH), 7.82–6.73 (m, 8H, ArH), 4.15 (d, *J* = 2.2 Hz, 1H, CH), 4.04 (q, *J* = 7.0 Hz, 2H, $CH_3\underline{CH_2}O$), 3.42 (d, *J* = 2.2 Hz, 1H, CH), 2.06 (s, 3H, CH_3), 1.80 (s, 3H, CH_3), 1.34 (t, *J* = 7.0 Hz, 3H, $C\underline{H_3}CH_2O$) ppm. ^{13}C NMR (DMSO-d_6) *δ* 167.45, 165.58, 156.74, 152.93, 149.46, 129.26, 127.85, 127.51, 127.01, 125.23, 122.67, 120.60 (2C), 116.44, 112.61, 93.04, 85.56, 64.37, 46.14, 31.90, 25.10, 15.06, 10.06 ppm. MS (EI, 70 eV): *m/z* (%) = 405 (36) [M$^+$], 253 (10), 137 (100), 108 (21). Anal. Calcd. for $C_{23}H_{23}N_3O_4$: C 68.13, H 5.72, N 10.36. Found: C 68.16, H 5.80, N 10.34%.

General procedure for the preparation of *N*-aryl-4-(2-hydroxyphenyl)-3,6-dimethylisoxazolo[5,4-*b*]pyridine-5-carboxamides 9e-g. The mixture containing 1 mmol of 3-methylisoxazol-5-amine (**1**), 1 mmol of salicylaldehyde (**2**), 1 mmol of the appropriate *N*-aryl-3-oxobutanamide **3e-g**, 0.05 mmol of ytterbium triflate and 3 mL of ethanol was continuously stirred at room temperature for 48 h. The solid precipitated was collected by

filtration and vacuum dried. The target compound was obtained in high purity (ca. 95% according to TLC and NMR) and no additional recrystallization was needed.

***N*-(2-Chlorophenyl)-4-(2-hydroxyphenyl)-3,6-dimethylisoxazolo[5,4-*b*]pyridine-5-carboxamide (9e).** Yield 72%, yellow solid, m.p. 215–216 °C. ^{1}H NMR (DMSO-d_6) δ 10.10 (s, 1H, NH), 9.81 (s, 1H, OH), 7.46–6.82 (m, 8H, ArH), 2.73 (s, 3H, CH_3), 2.03 (s, 3H, CH_3) ppm. ^{13}C NMR (DMSO-d_6) δ 168.50, 165.95, 158.22, 156.86, 155.10, 143.08, 134.67, 131.22, 130.71, 130.54, 130.04, 128.27, 127.80, 127.61, 127.24, 120.83, 119.26, 116.02, 110.34, 23.49, 11.24 ppm. MS (EI, 70 eV): *m/z* (%) = 395 (7), 393 (22) [M^+], 267 (100), 129 (26), 127 (82). Anal. Calcd. for $C_{21}H_{16}ClN_3O_3$: C 64.05, H 4.09, N 10.67. Found: C 64.02, H 4.11, N 10.65%.

4-(2-Hydroxyphenyl)-3,6-dimethyl-*N*-(*o*-tolyl)isoxazolo[5,4-*b*]pyridine-5-carboxamide (9f). Yield 77%, yellow solid, m.p. 234–235 °C. ^{1}H NMR (DMSO-d_6) δ 9.85 (s, 1H, OH), 9.79 (s, 1H, NH), 7.35–6.85 (m, 8H, ArH), 2.73 (s, 3H, CH_3), 2.03 (s, 3H, CH_3), 1.94 (s, 3H, CH_3) ppm. ^{13}C NMR (DMSO-d_6) δ 168.39, 165.65, 158.16, 156.82, 155.12, 142.85, 136.10, 133.07, 131.18 (2C), 130.78, 130.72, 126.34, 126.26, 125.76, 120.91, 119.21, 115.94, 110.31, 23.40, 17.94, 11.23 ppm. MS (EI, 70 eV): *m/z* (%) = 373 (12) [M^+], 267 (22), 107 (100). Anal. Calcd. for $C_{22}H_{19}N_3O_3$: C 70.76, H 5.13, N 11.25. Found: C 70.70, H 5.17, N 11.28%.

***N*-(2,4-Dimethylphenyl)-4-(2-hydroxyphenyl)-3,6-dimethylisoxazolo[5,4-*b*]pyridine-5-carboxamide (9g).** Yield 76%, yellow solid, m.p. 239–240 °C. ^{1}H NMR (DMSO-d_6) δ 9.83 (s, 1H, OH), 9.69 (s, 1H, NH), 7.34–6.83 (m, 7H, ArH), 2.71 (s, 3H, CH_3), 2.19 (s, 3H, CH_3), 2.02 (s, 3H, CH_3), 1.88 (s, 3H, CH_3) ppm. ^{13}C NMR (DMSO-d_6) δ 168.37, 165.63, 158.17, 156.82, 155.10, 142.82, 135.41, 133.50, 132.96, 131.26 (2C), 131.15, 130.73, 126.83, 125.72, 120.93, 119.20, 115.93, 110.30, 23.39, 20.92, 17.86, 11.22 ppm. MS (EI, 70 eV): *m/z* (%) = 387 (16) [M^+], 267 (29), 121 (100). Anal. Calcd. for $C_{23}H_{21}N_3O_3$: C 71.30, H 5.46, N 10.85. Found: C 71.38, H 5.41, N 10.81%.

References

1. Sheldrick, G. M. *Acta Crystallogr. Sect. A* **2008**, *A64*, 112–122.

Copies of ^{1}H and ^{13}C NMR spectra

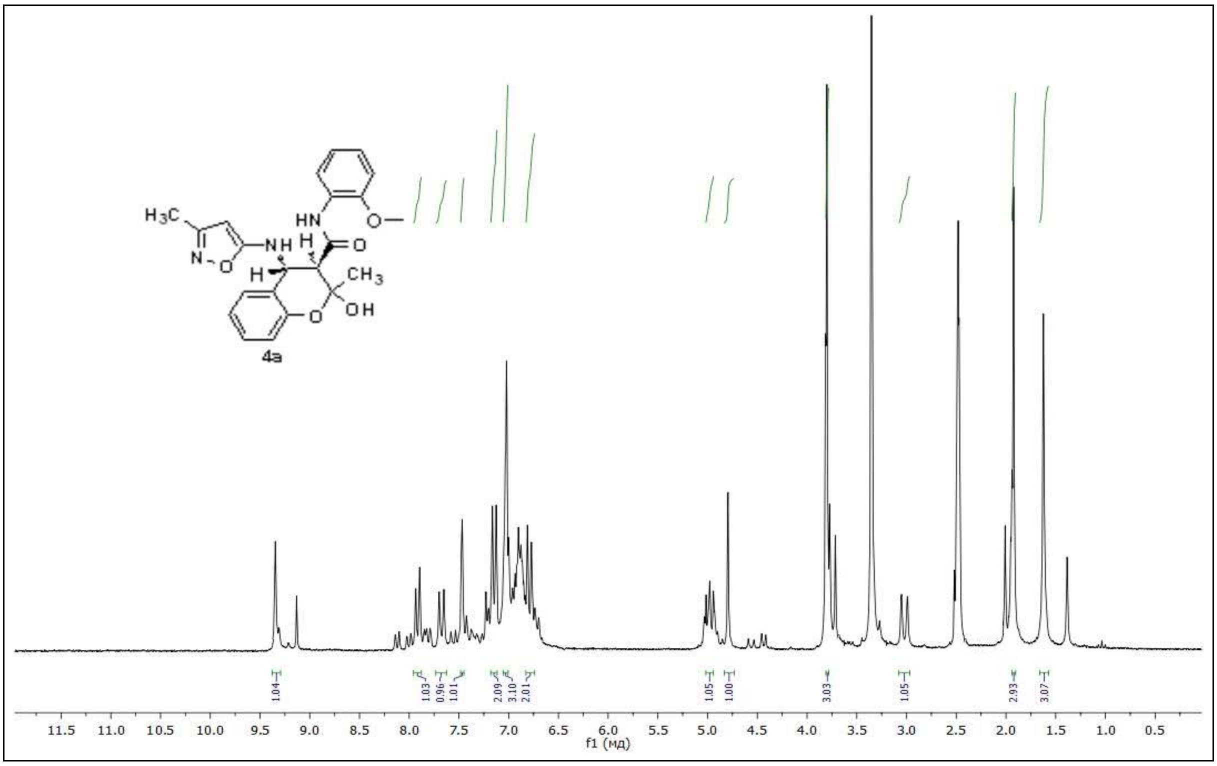

^{1}H NMR spectrum of compound **4a**

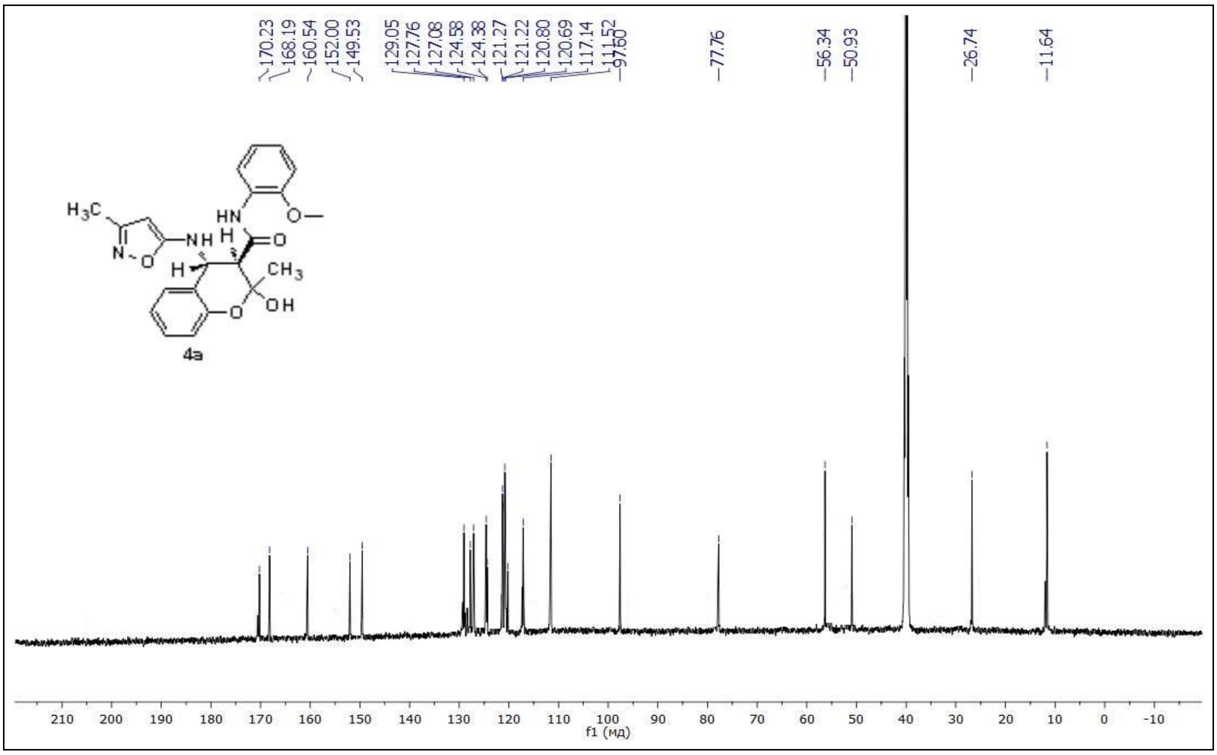

^{13}C NMR spectrum of compound **4a**

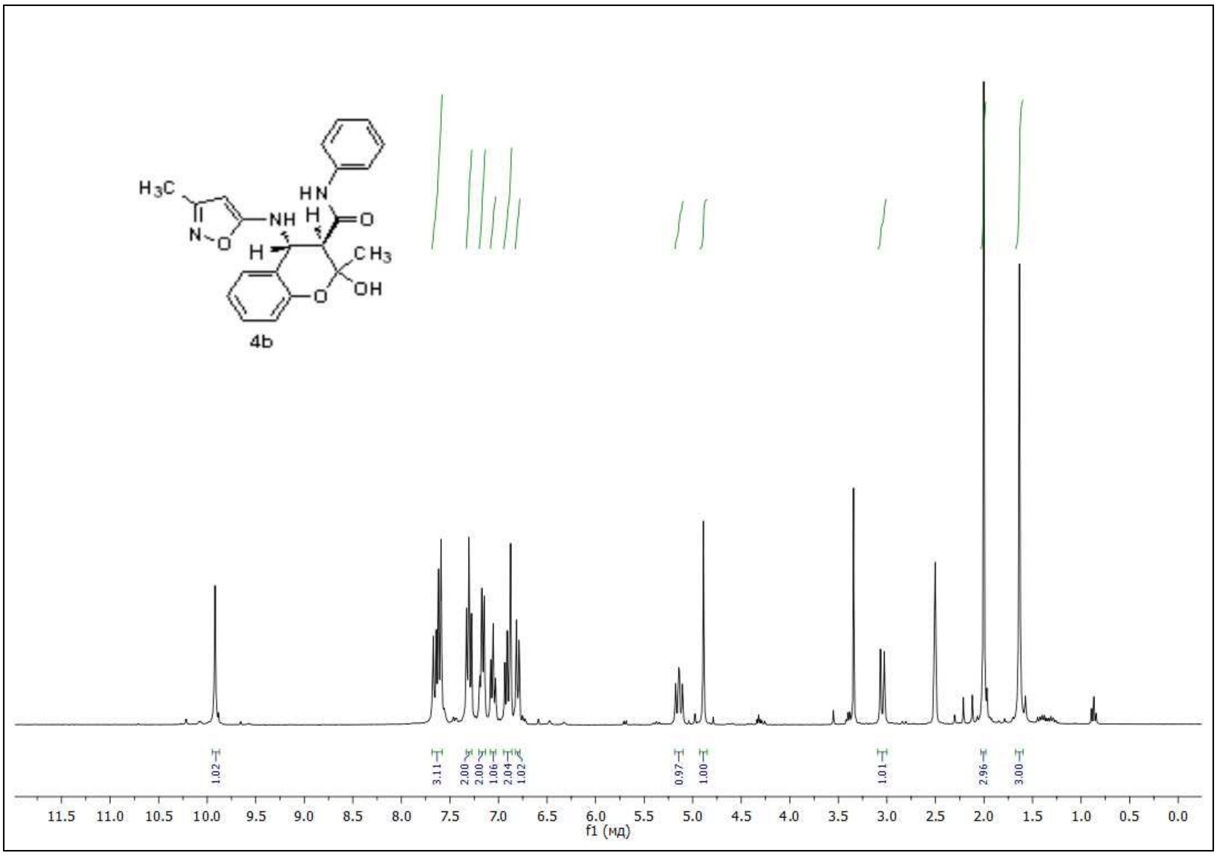

^{1}H NMR spectrum of compound **4b**

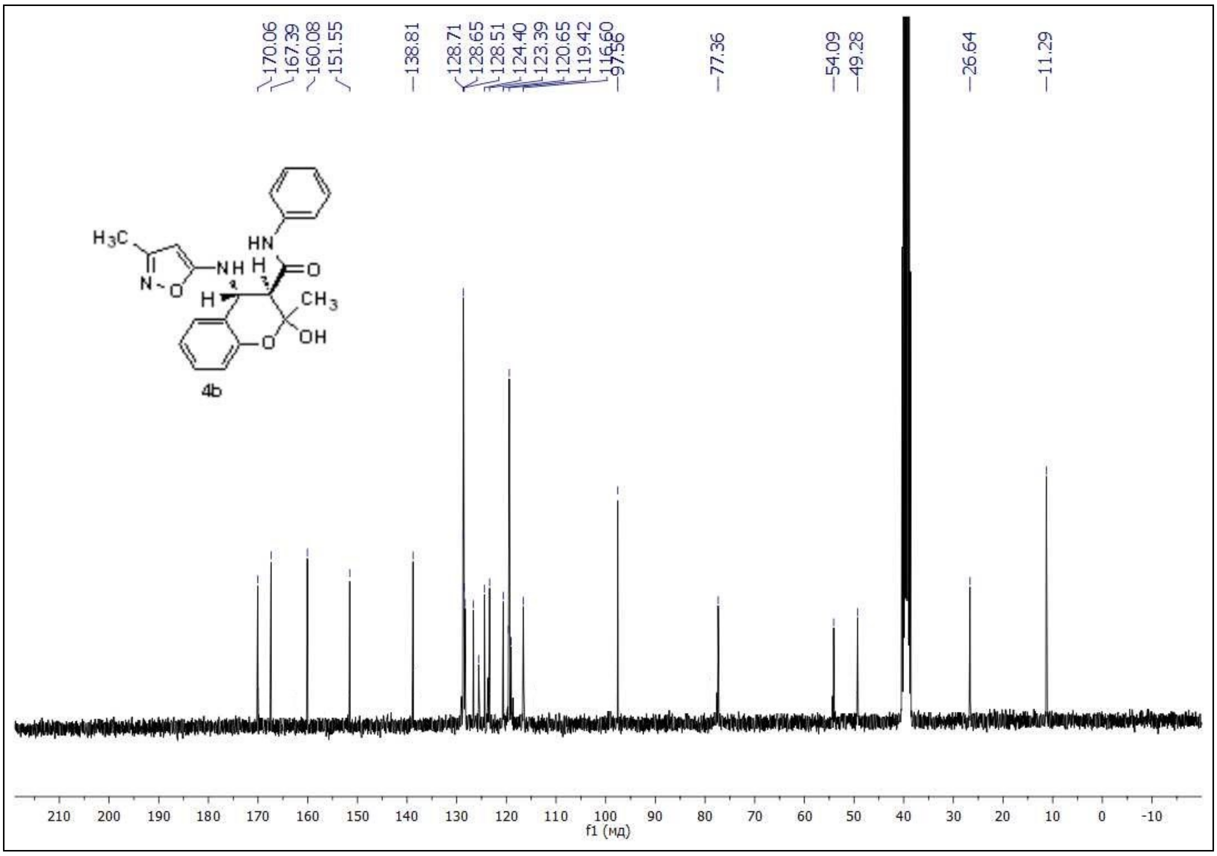

^{13}C NMR spectrum of compound **4b**

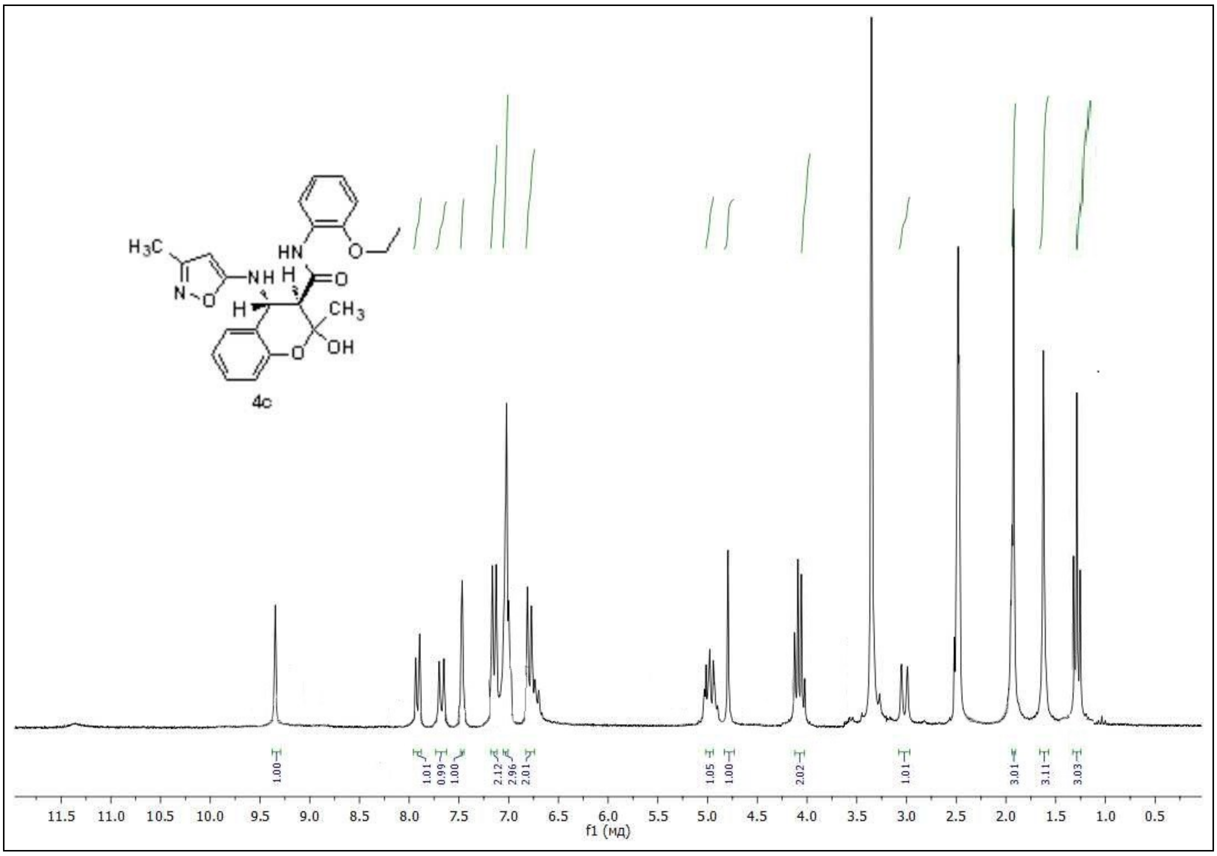

^{1}H NMR spectrum of compound **4c**

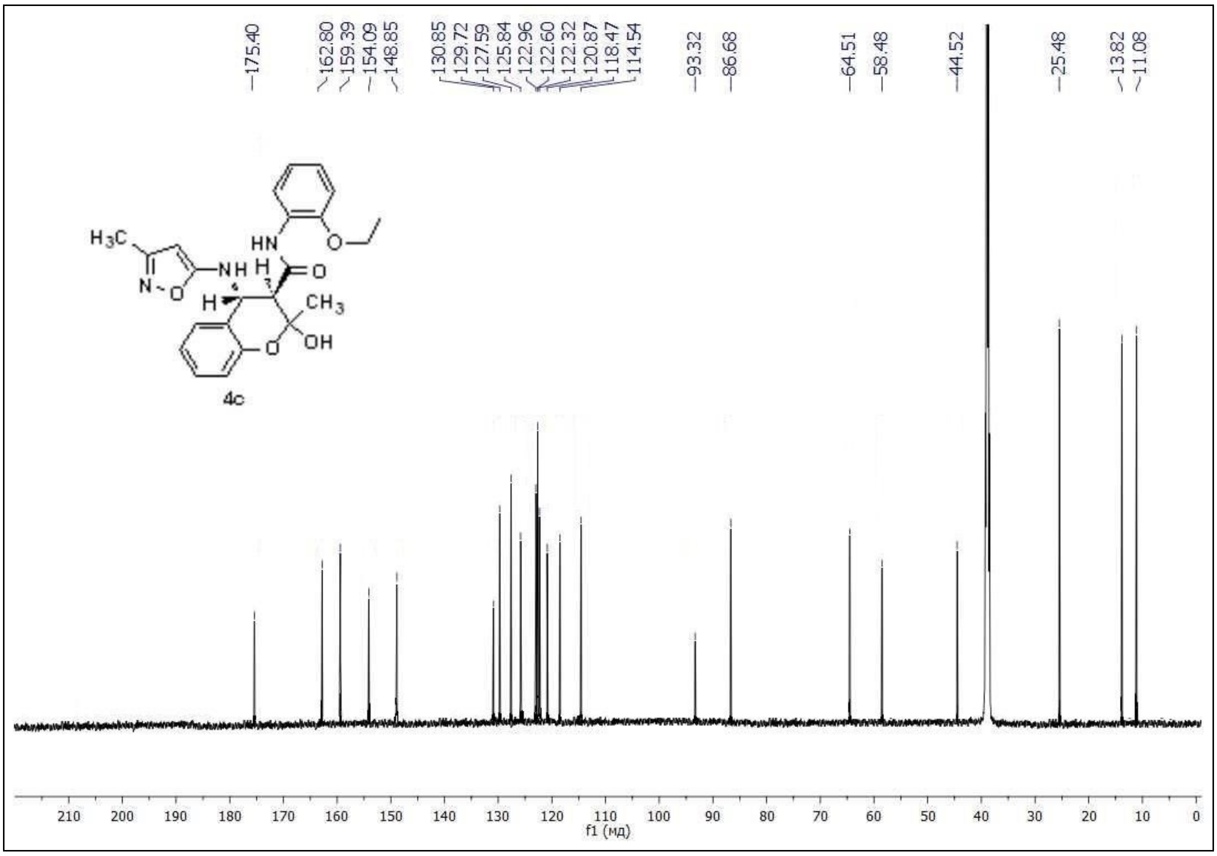

^{13}C NMR spectrum of compound **4c**

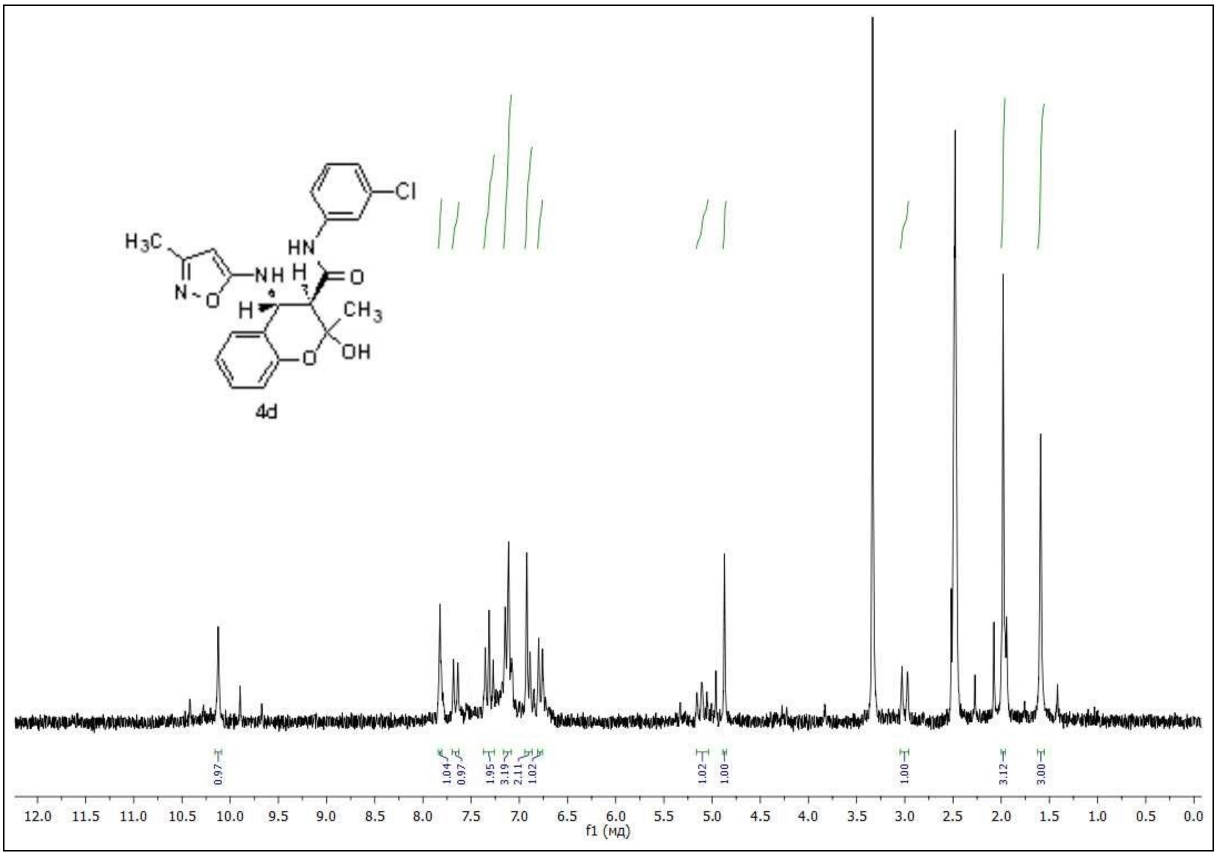

^{1}H NMR spectrum of compound **4d**

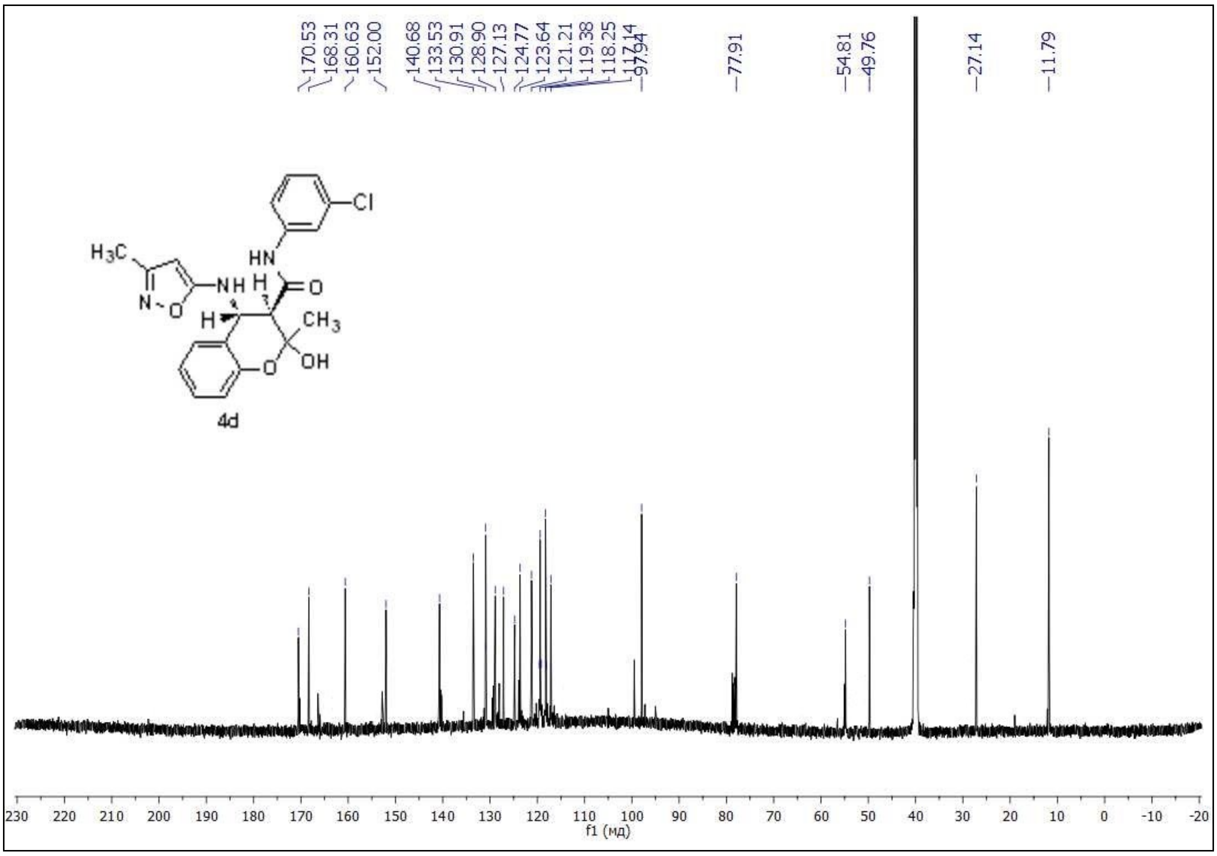

^{13}C NMR spectrum of compound **4d**

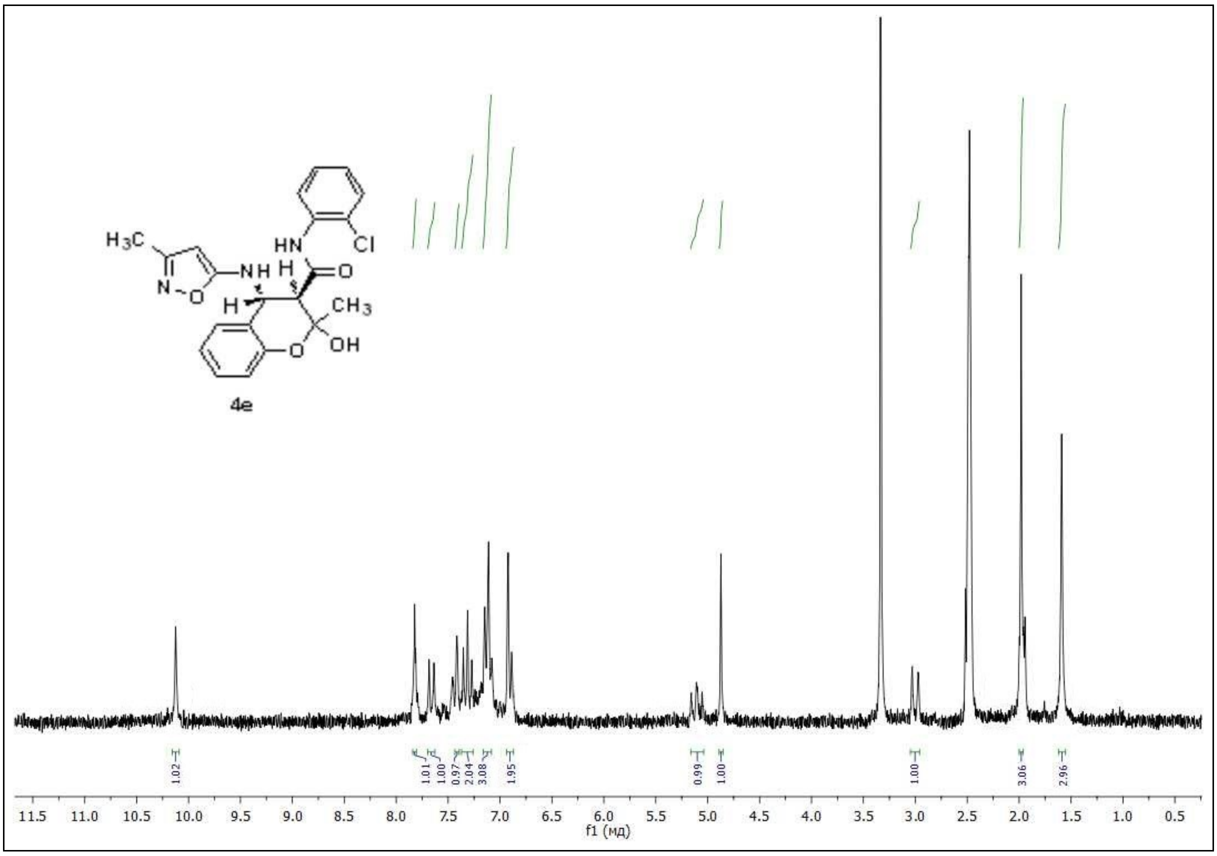

^{1}H NMR spectrum of compound **4e**

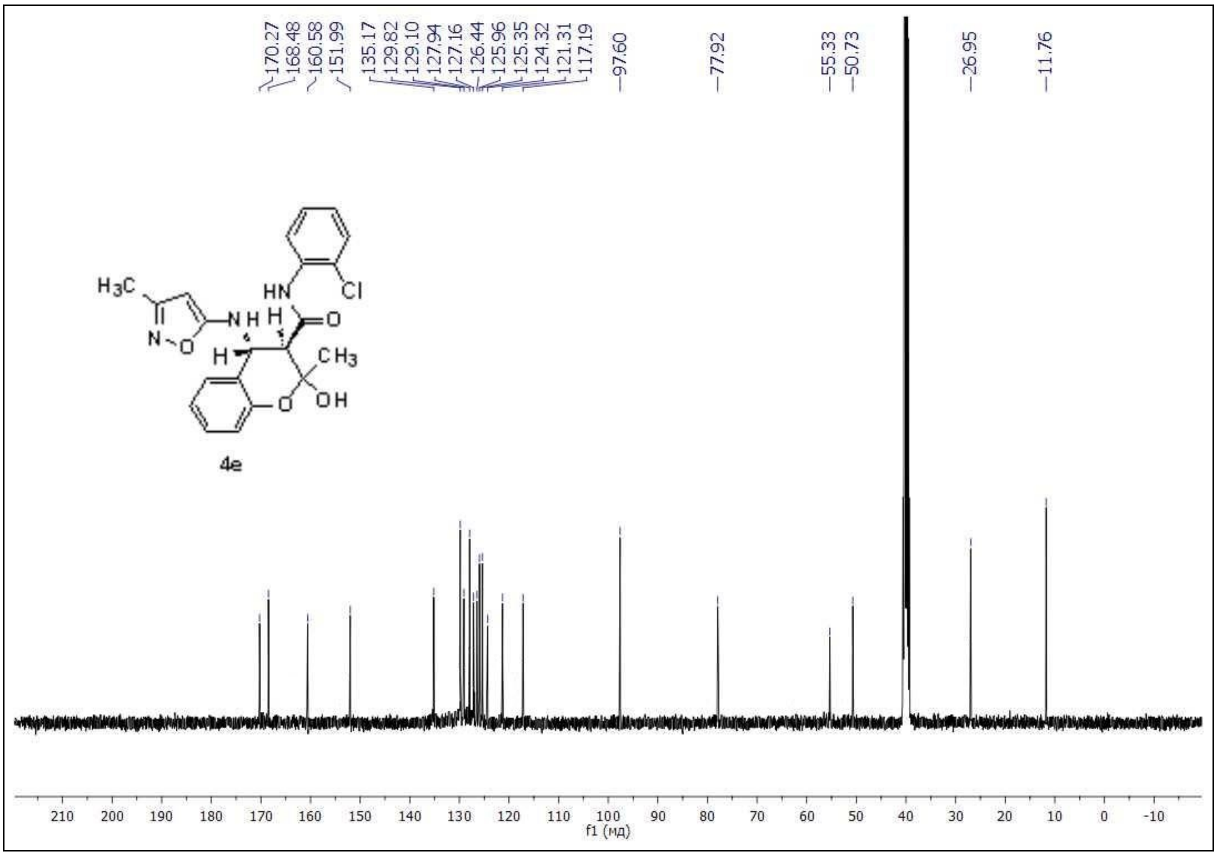

^{13}C NMR spectrum of compound **4e**

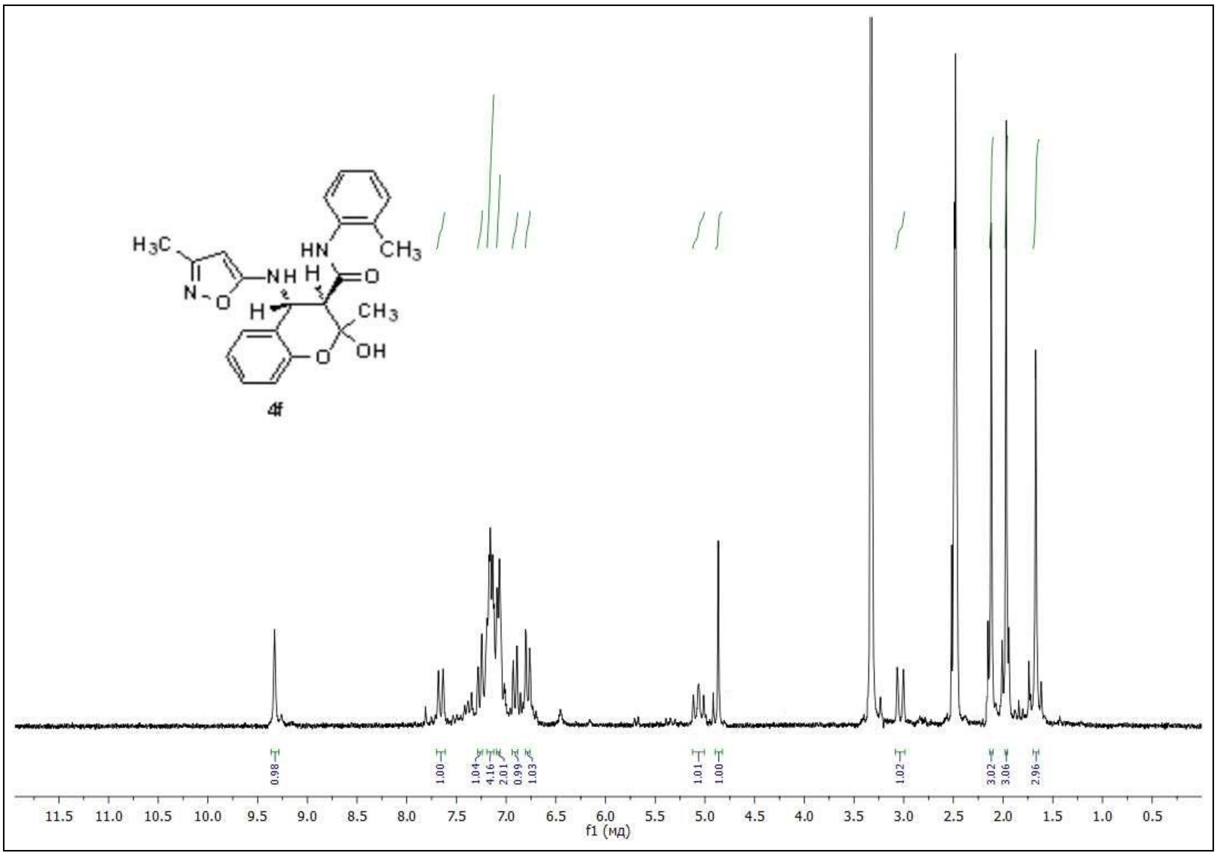

^{1}H NMR spectrum of compound **4f**

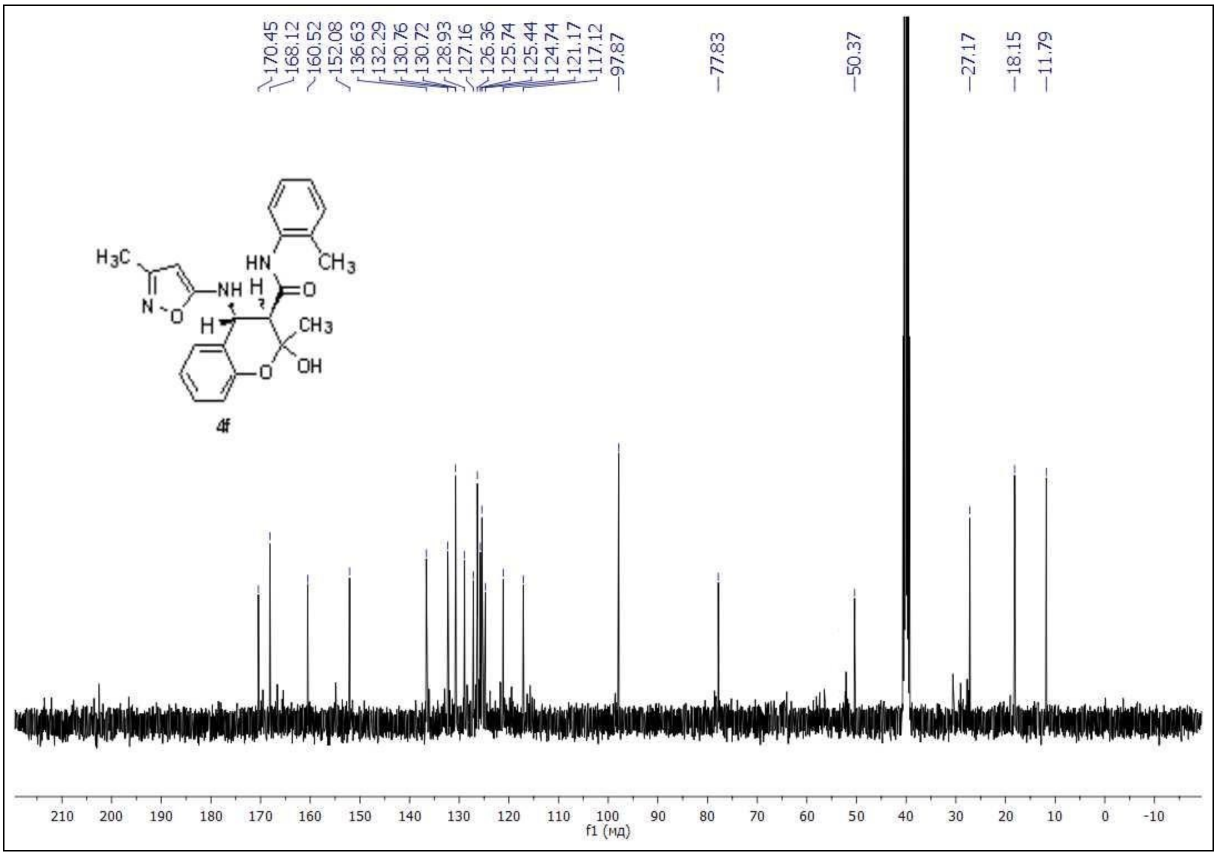

^{13}C NMR spectrum of compound **4f**

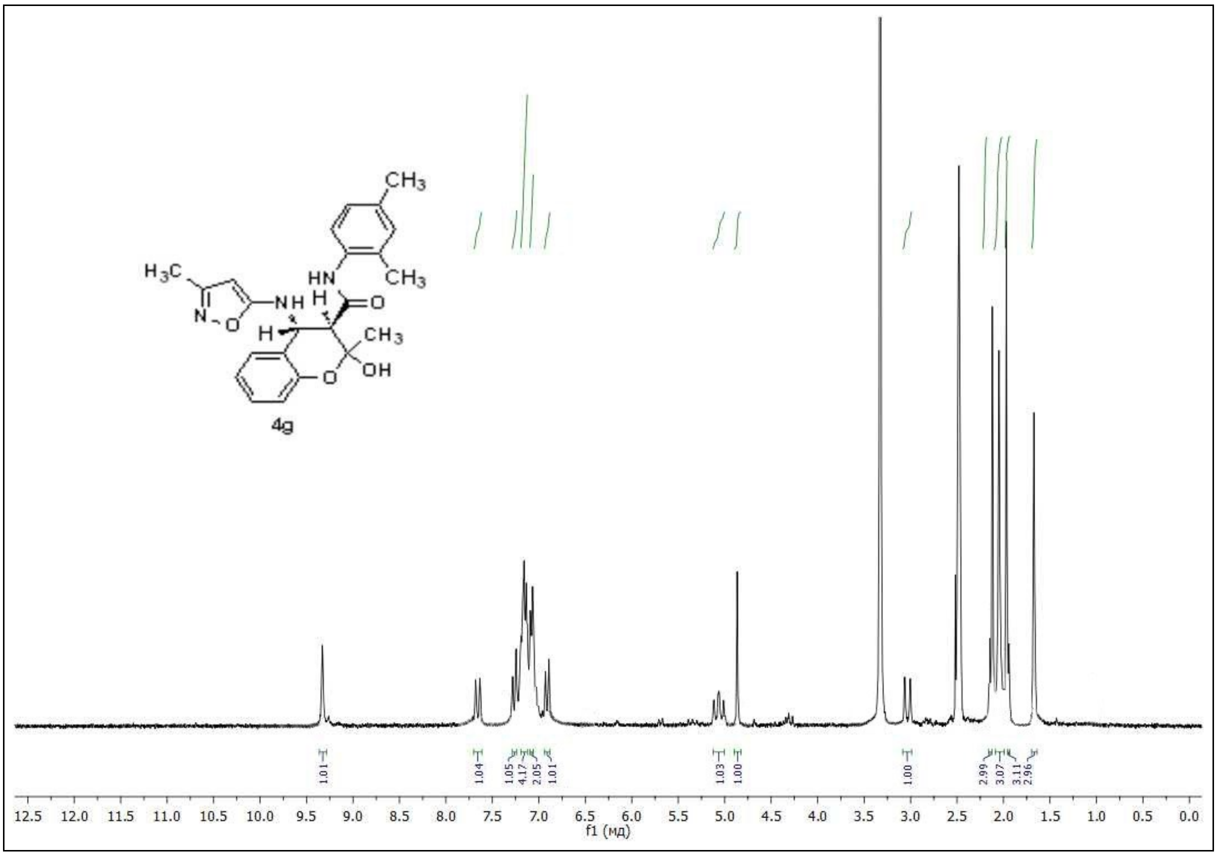

^{1}H NMR spectrum of compound **4g**

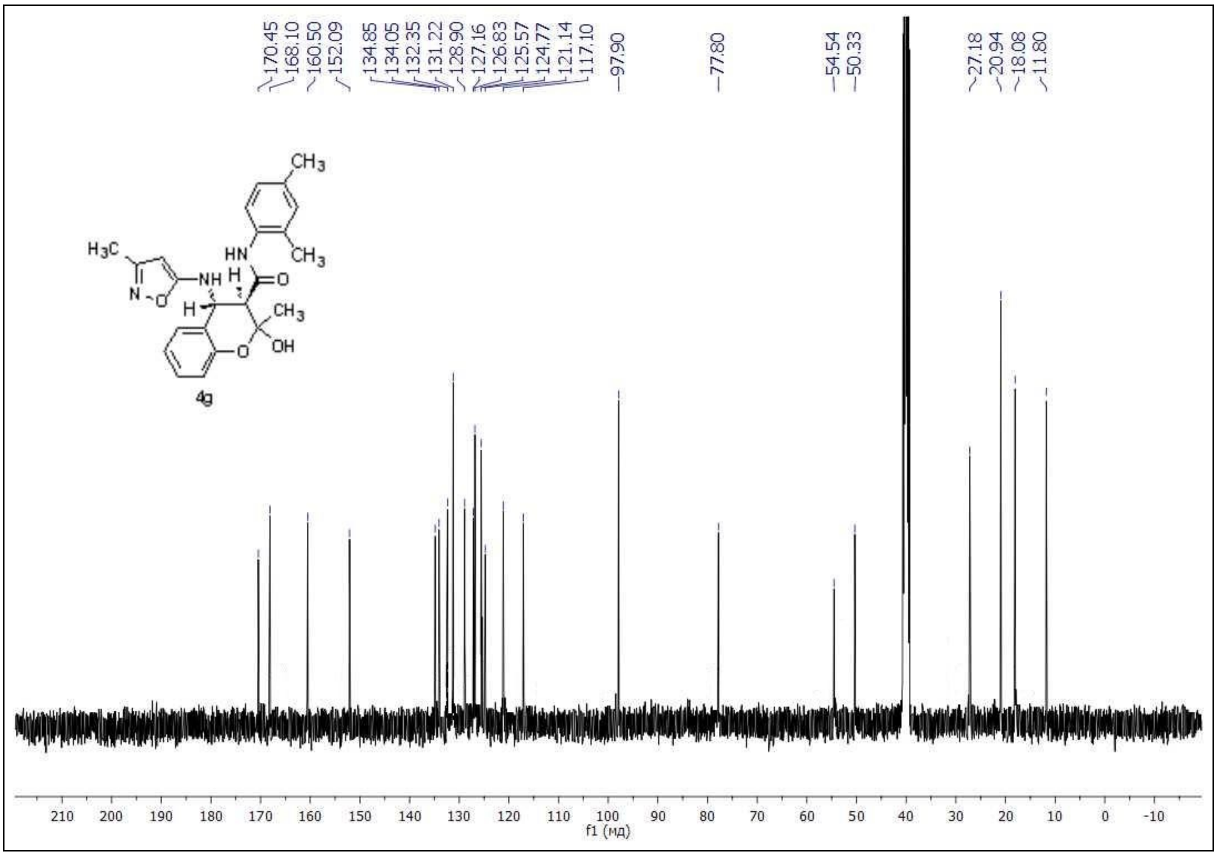

^{13}C NMR spectrum of compound **4g**

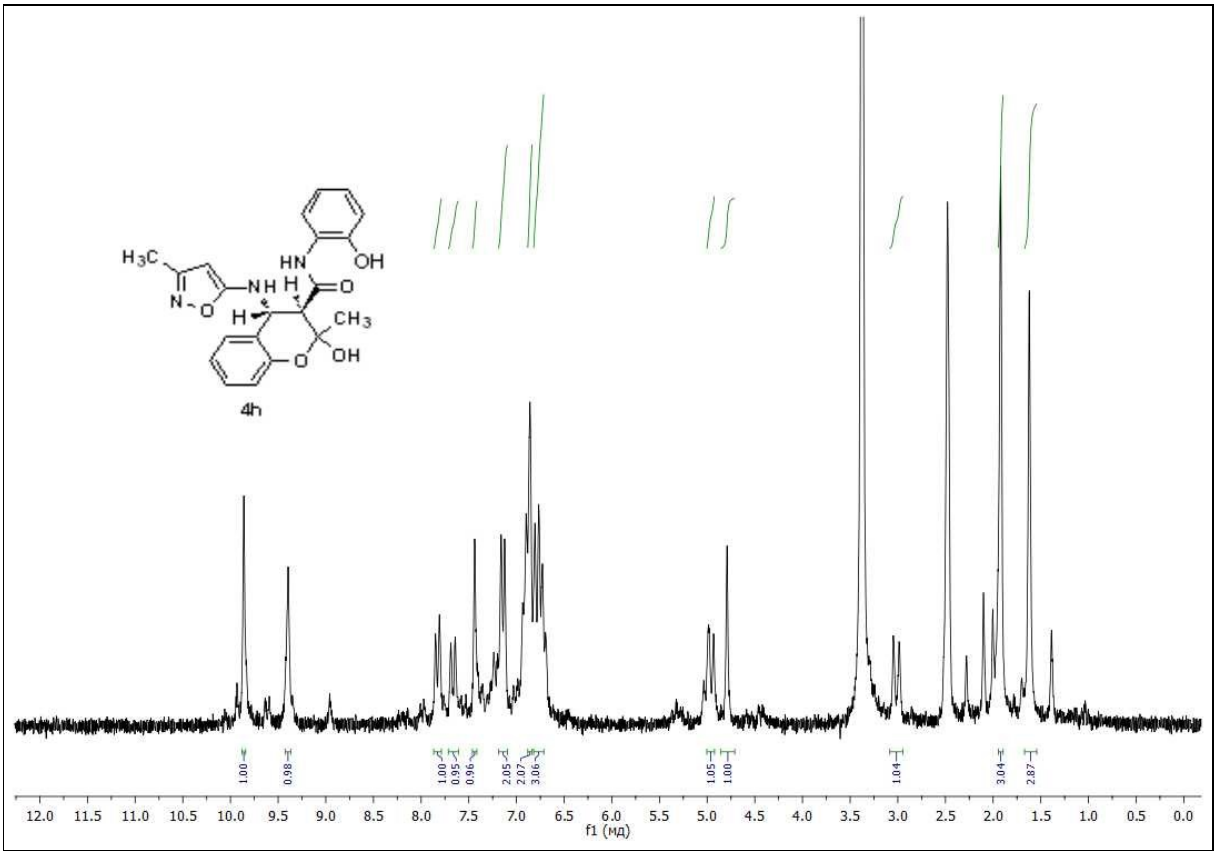

^{1}H NMR spectrum of compound **4h**

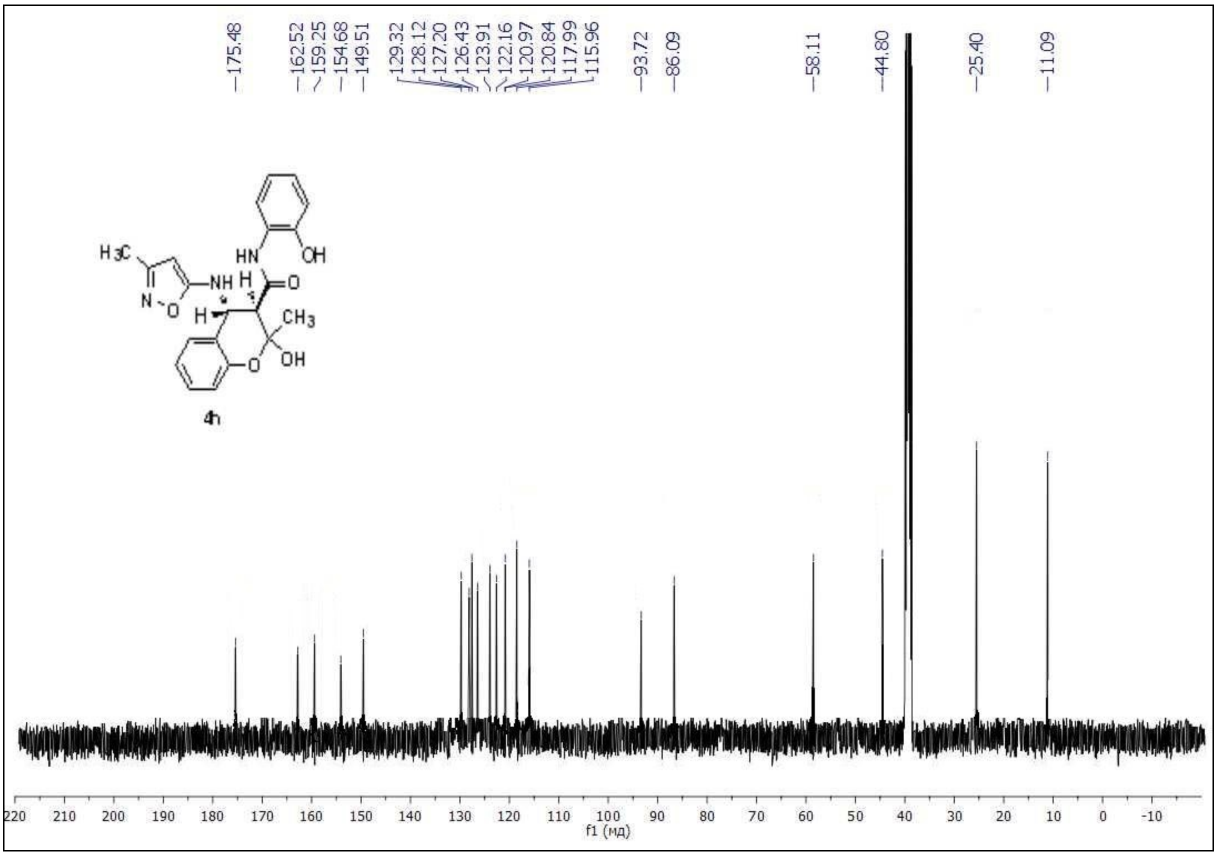

^{13}C NMR spectrum of compound **4h**

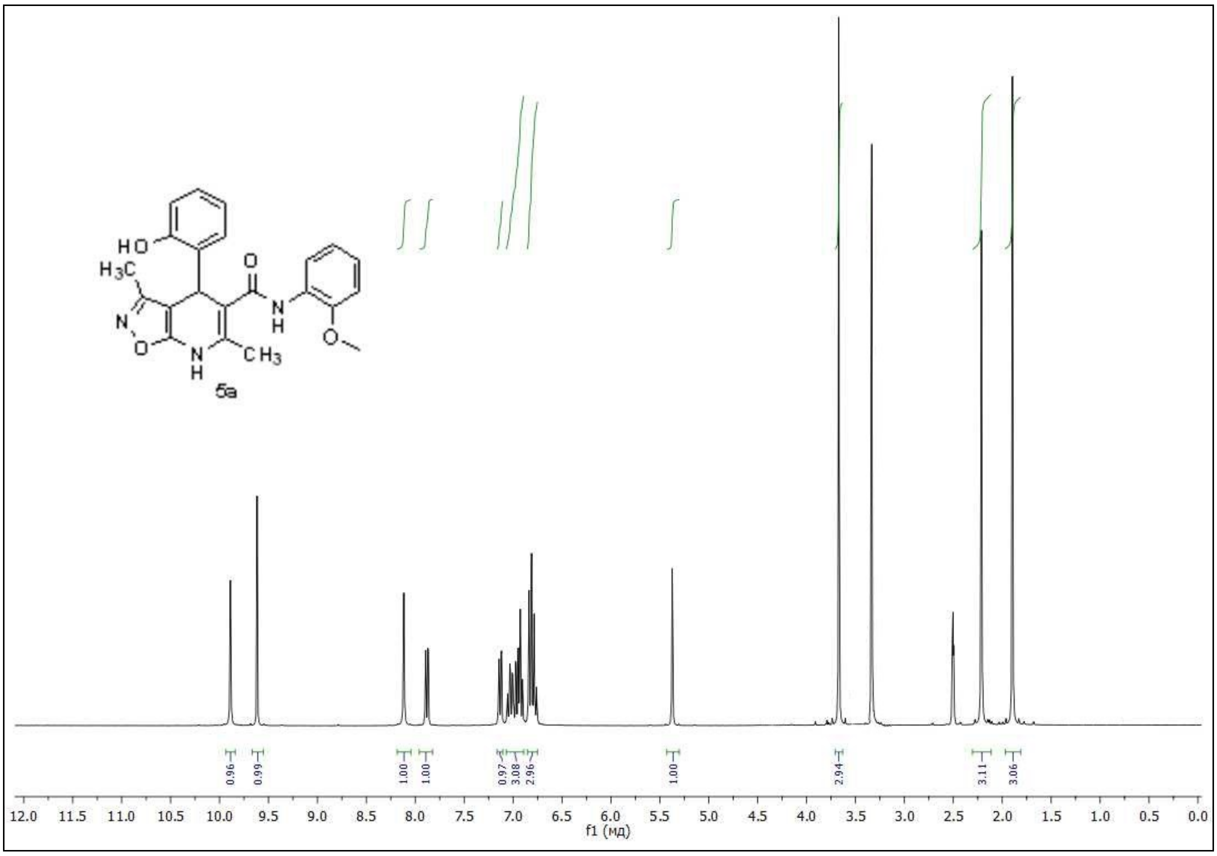

^{1}H NMR spectrum of compound **5a**

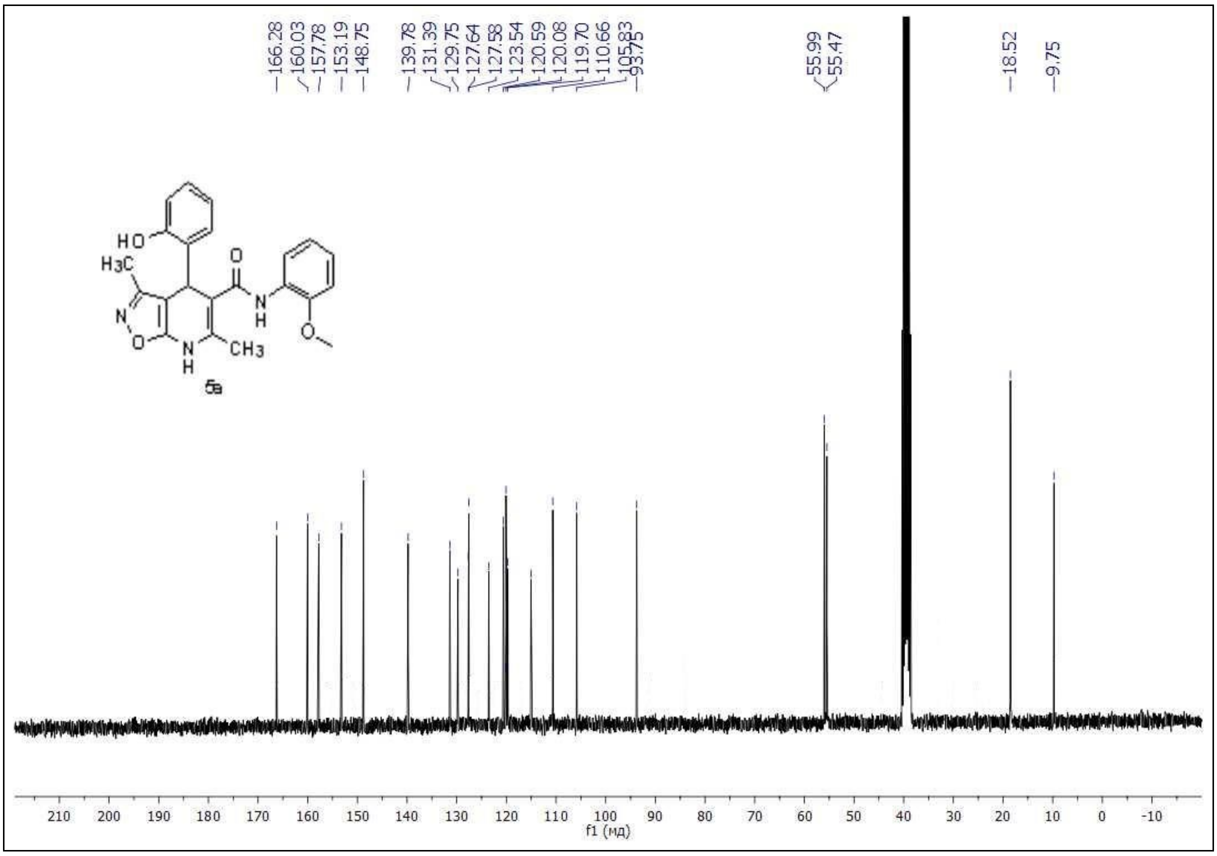

^{13}C NMR spectrum of compound **5a**

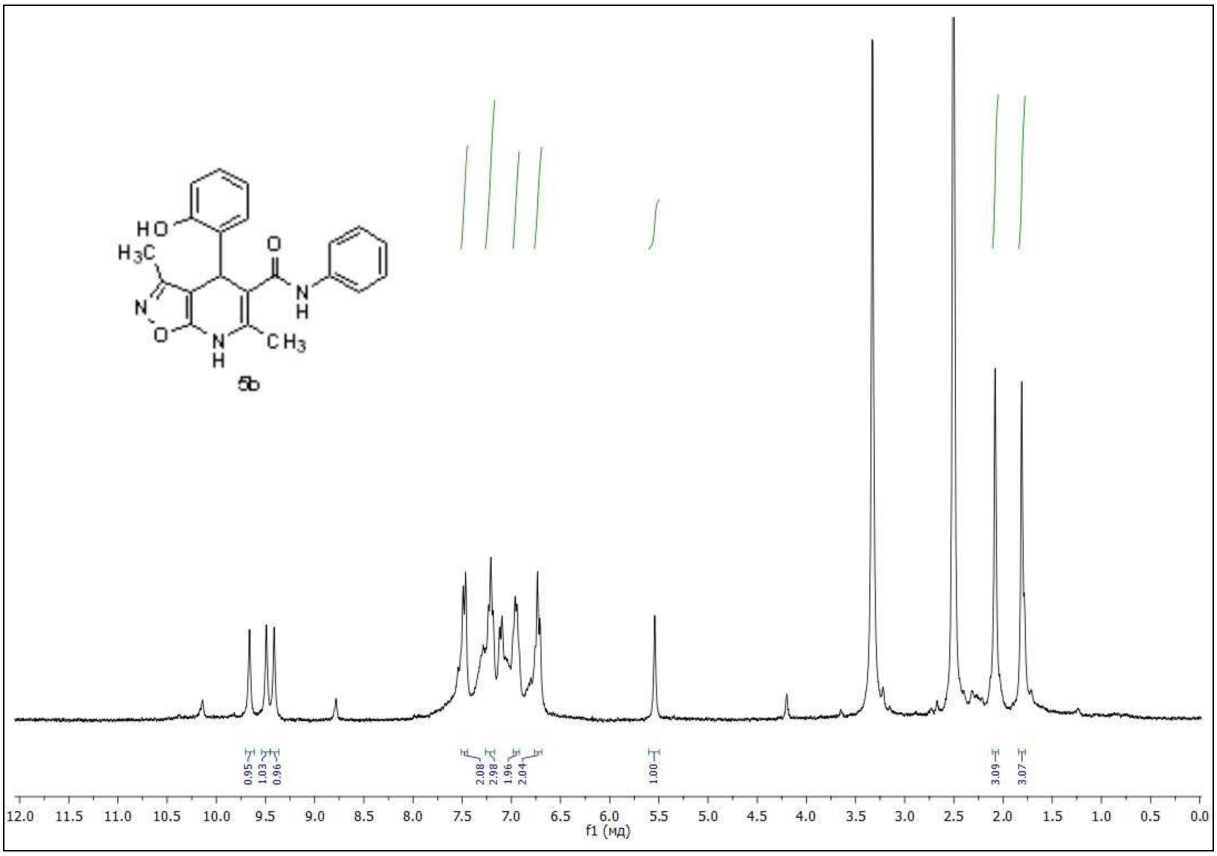

^{1}H NMR spectrum of compound **5b**

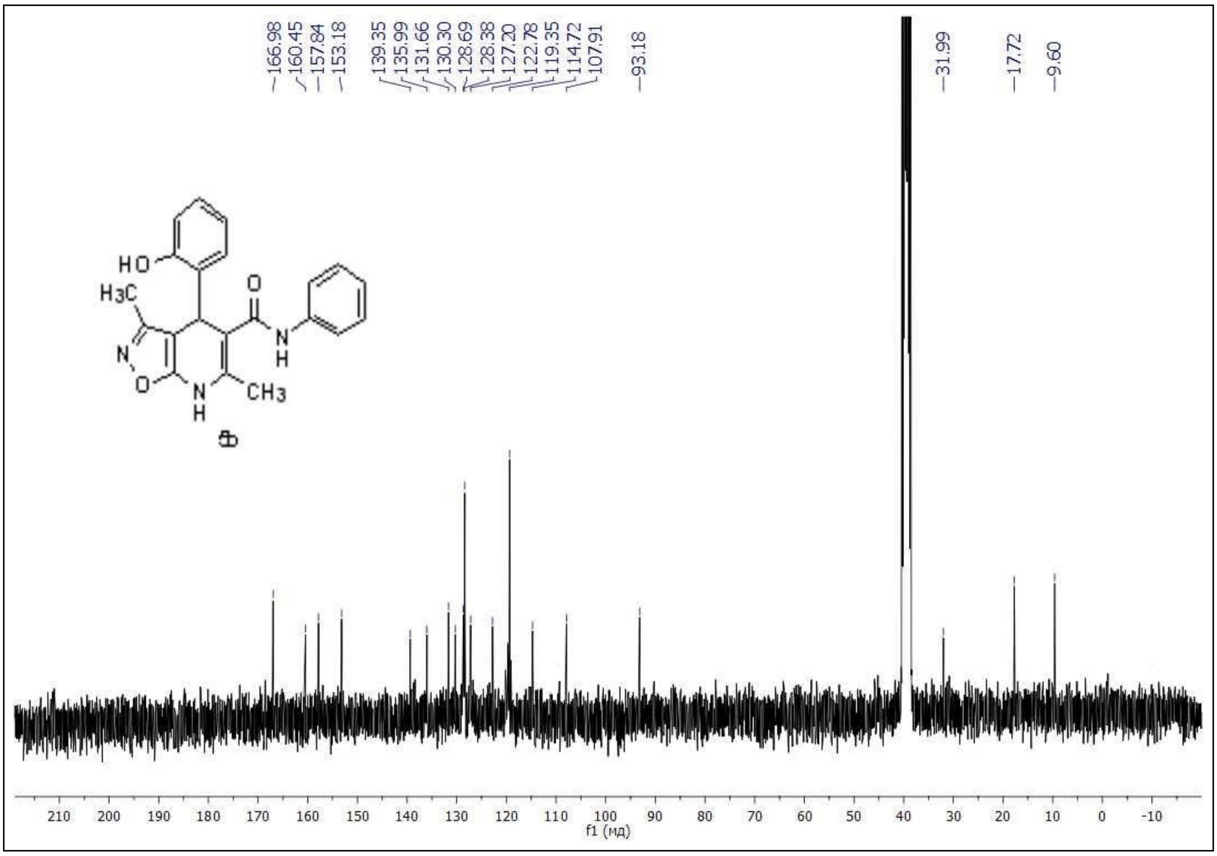

^{13}C NMR spectrum of compound **5b**

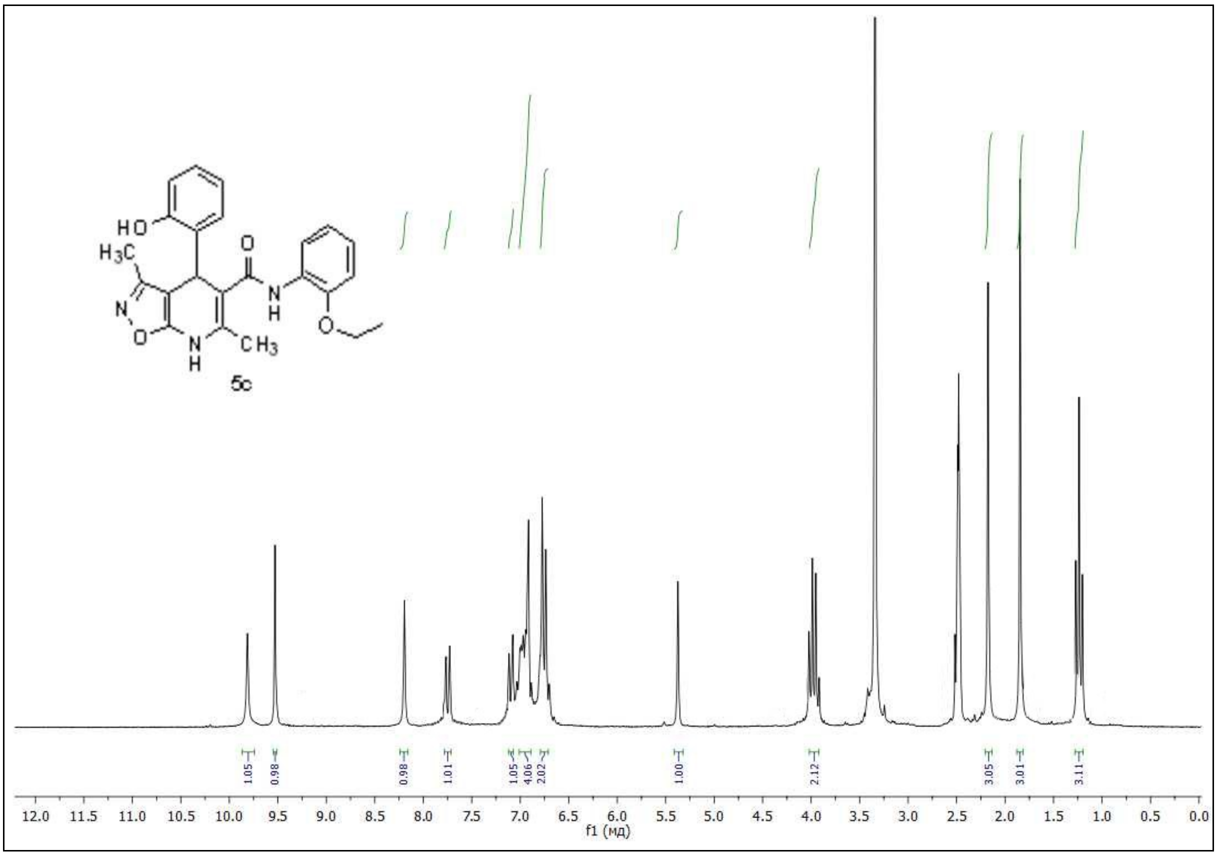

^{1}H NMR spectrum of compound **5c**

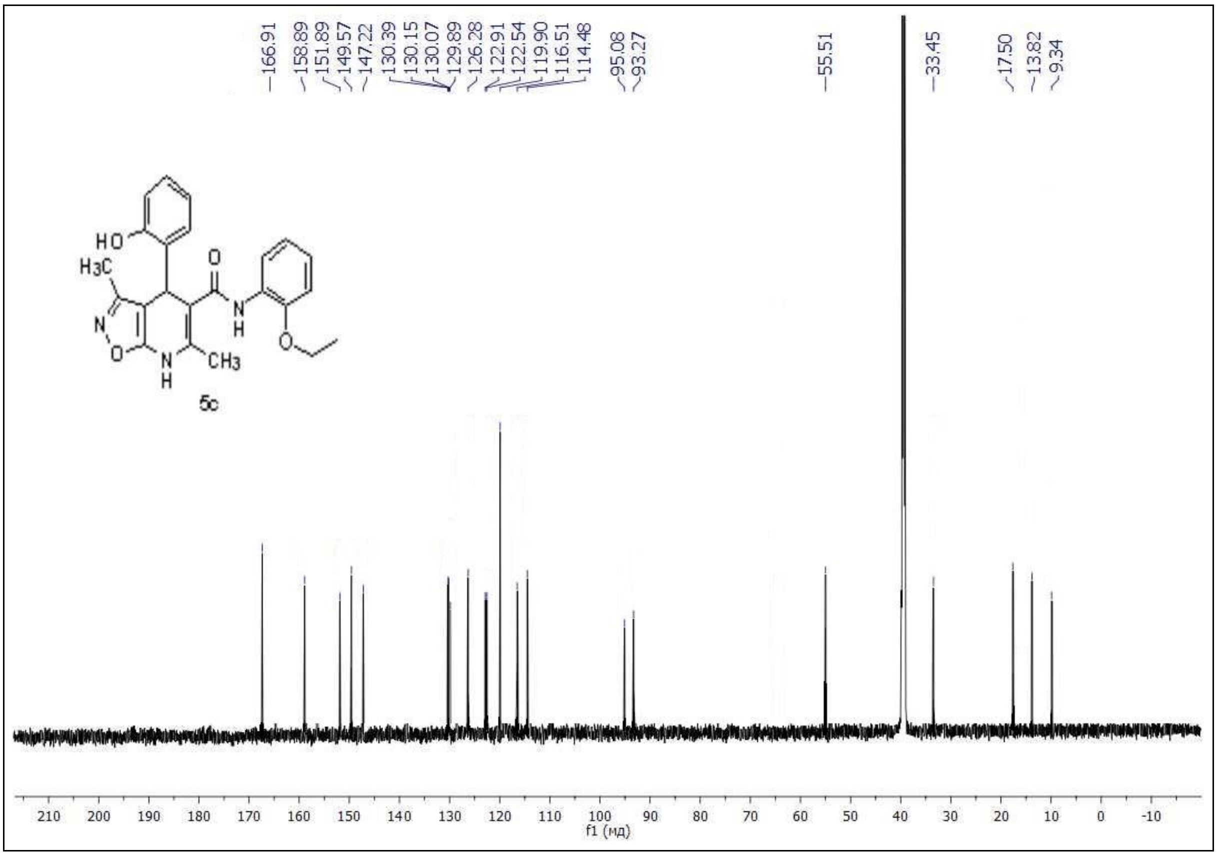

^{13}C NMR spectrum of compound **5c**

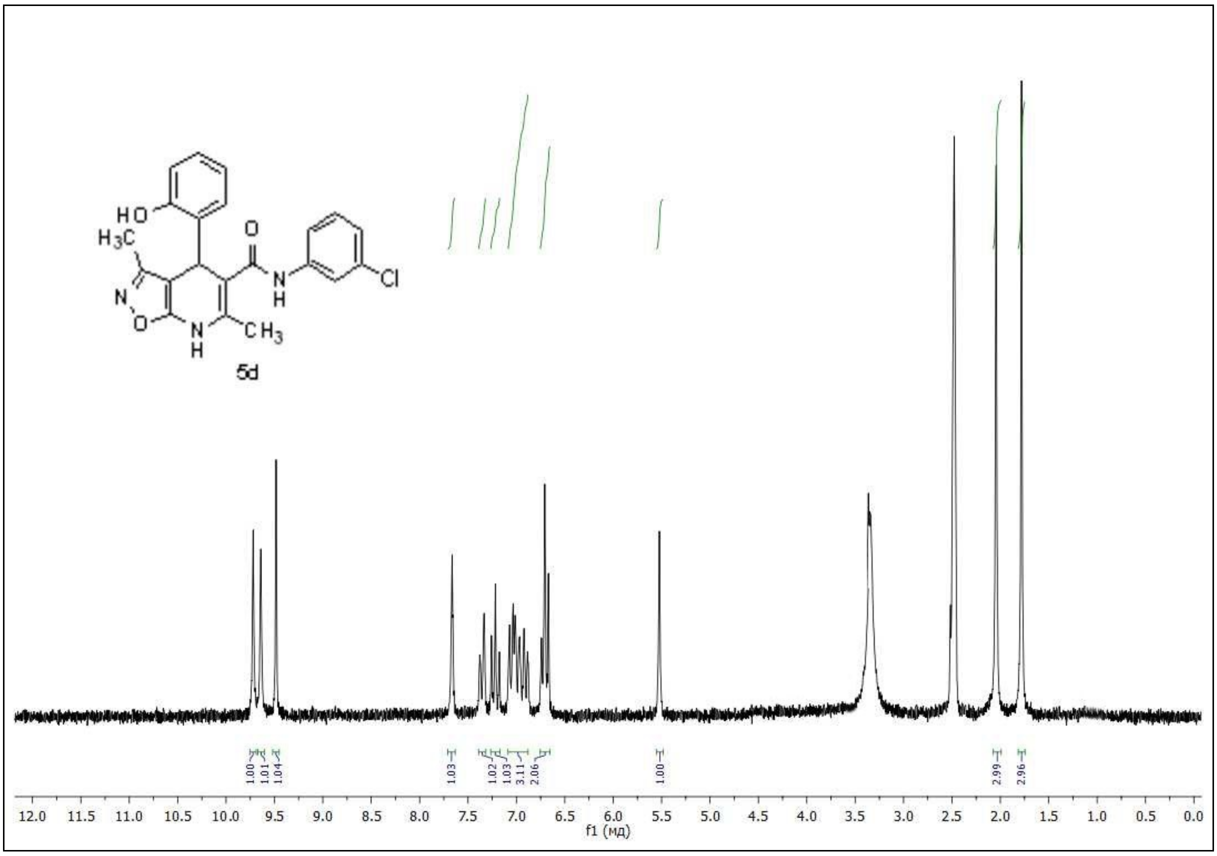

^{1}H NMR spectrum of compound **5d**

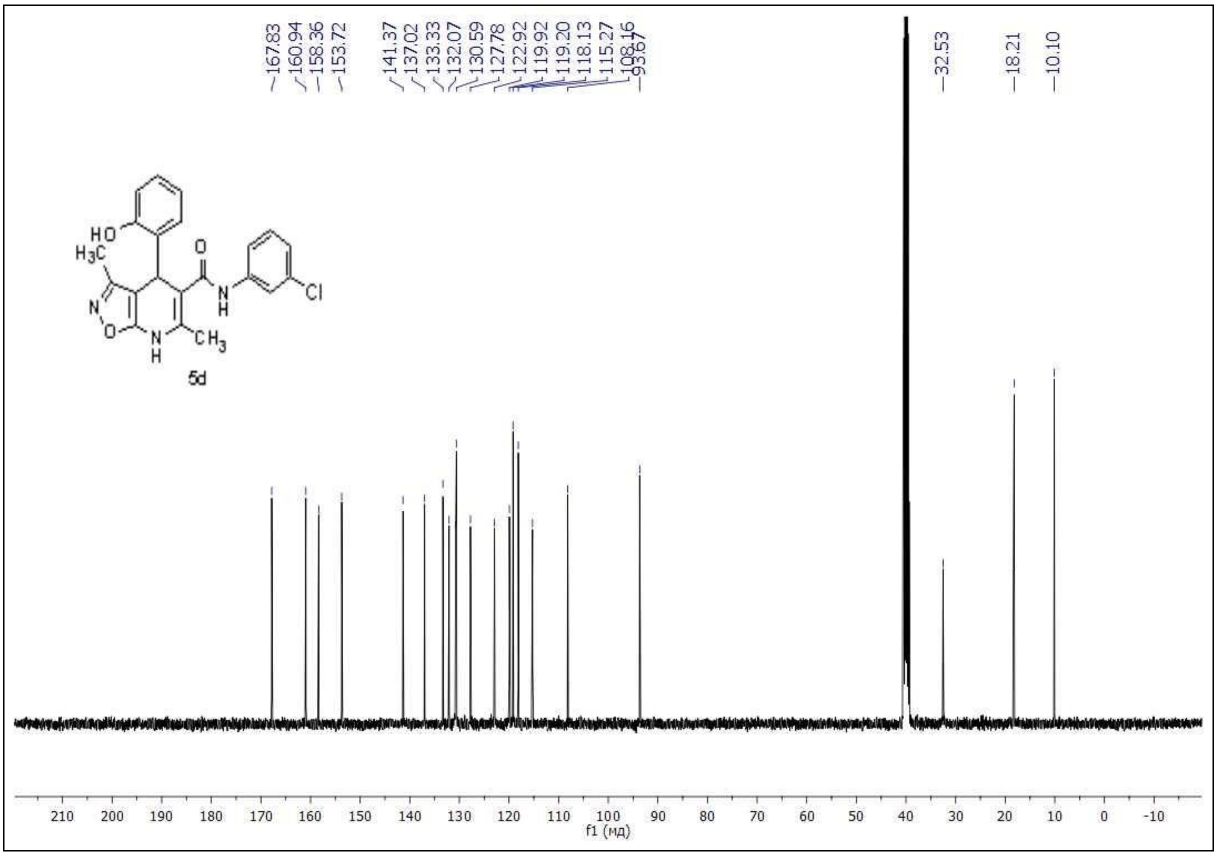

^{13}C NMR spectrum of compound **5d**

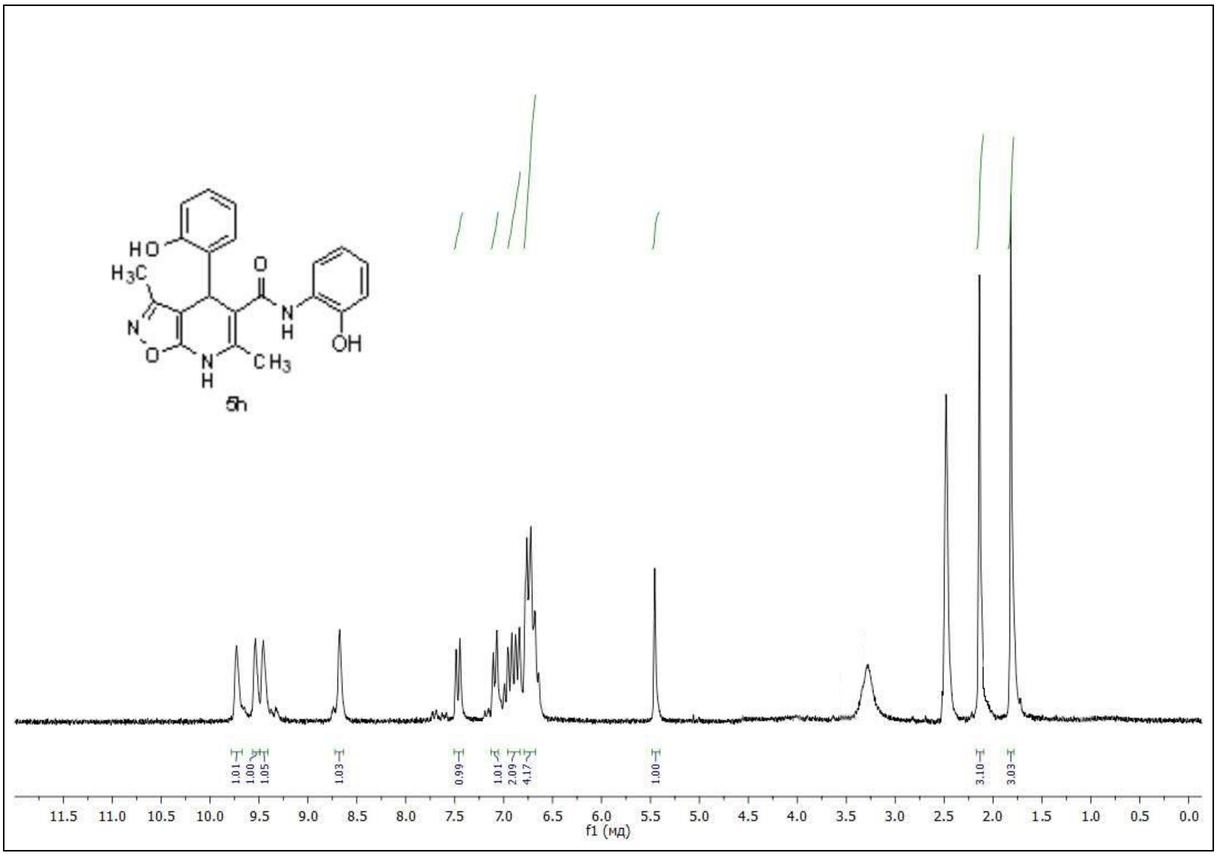

^{1}H NMR spectrum of compound **5h**

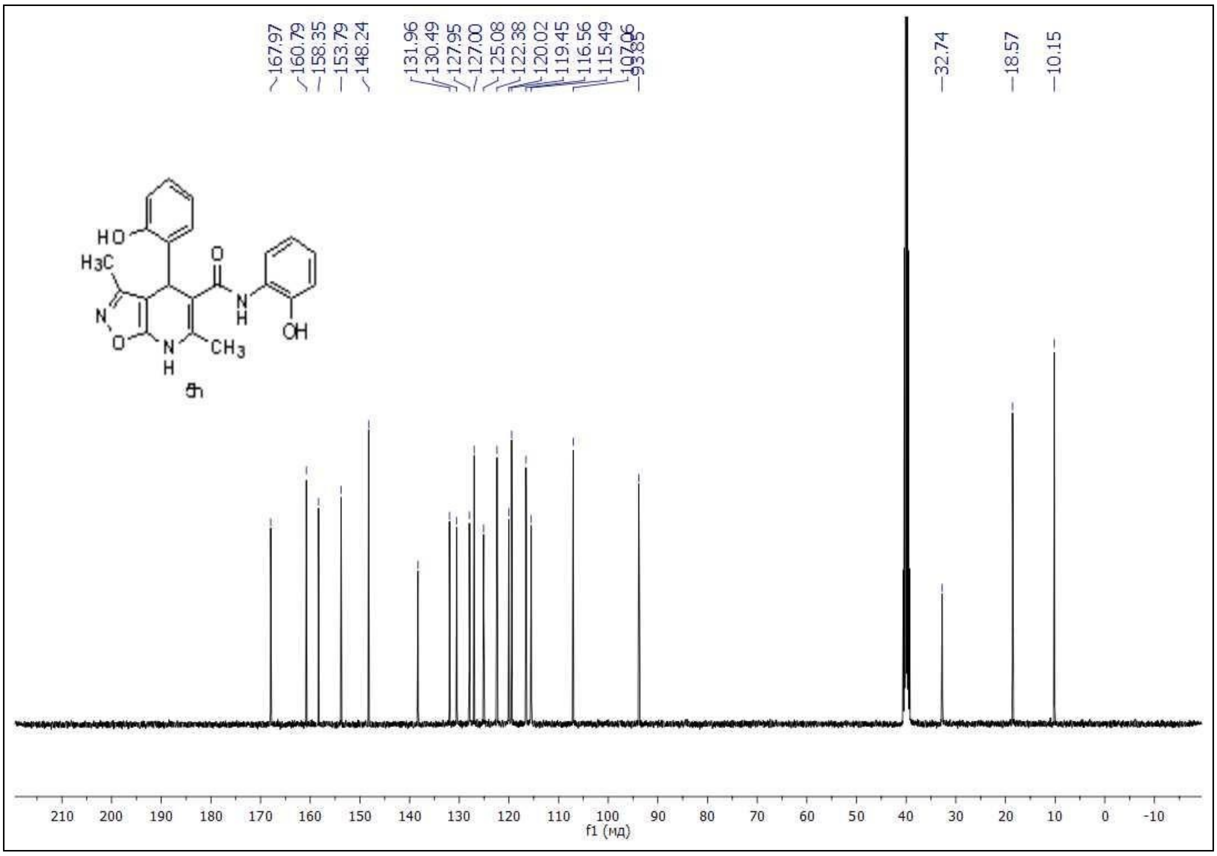

^{13}C NMR spectrum of compound **5h**

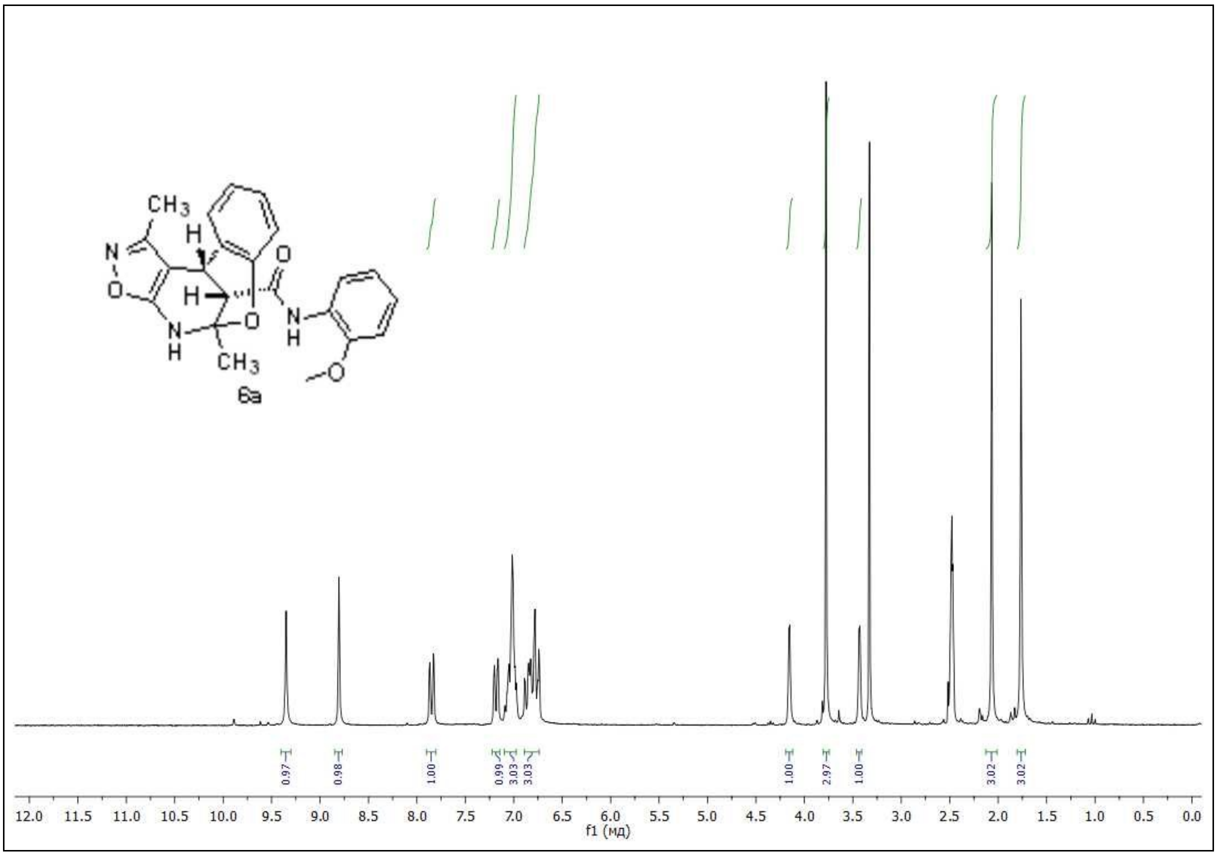

^{1}H NMR spectrum of compound **6a**

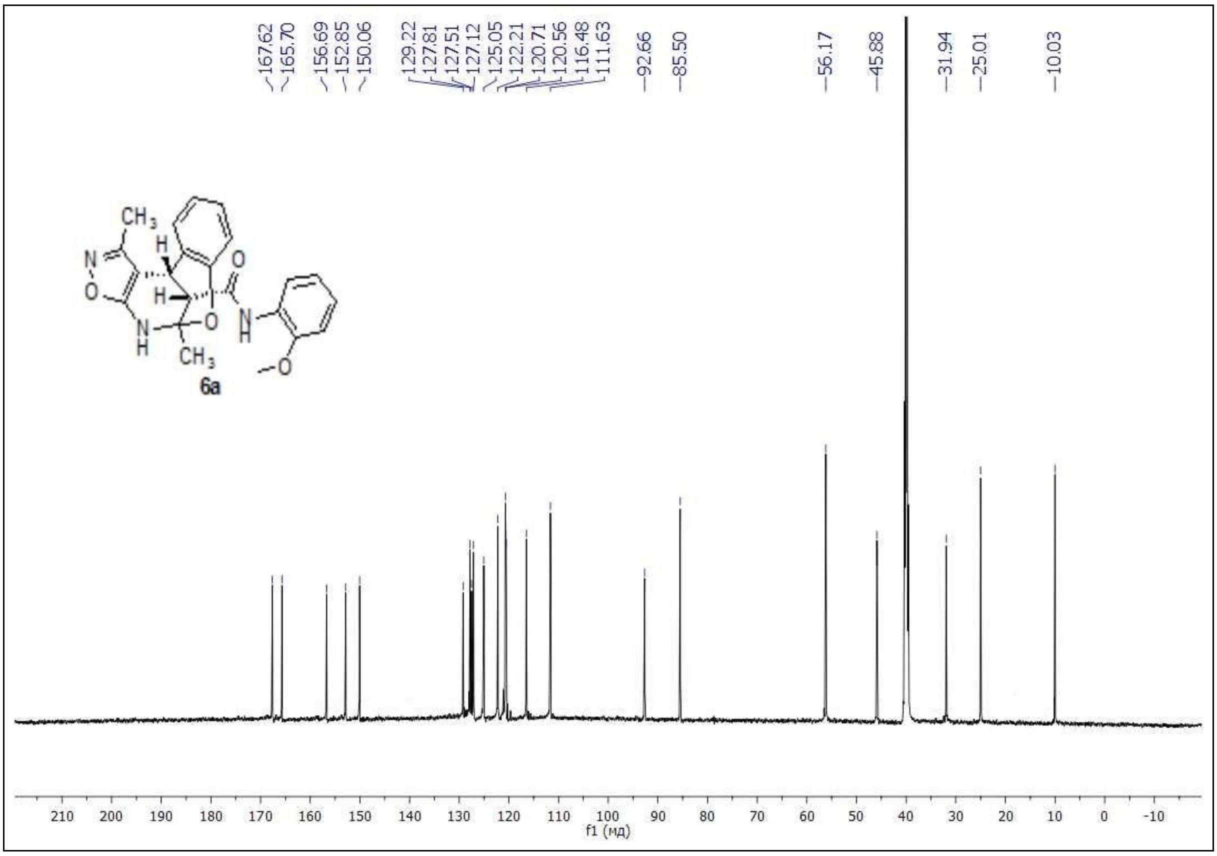

^{13}C NMR spectrum of compound **6a**

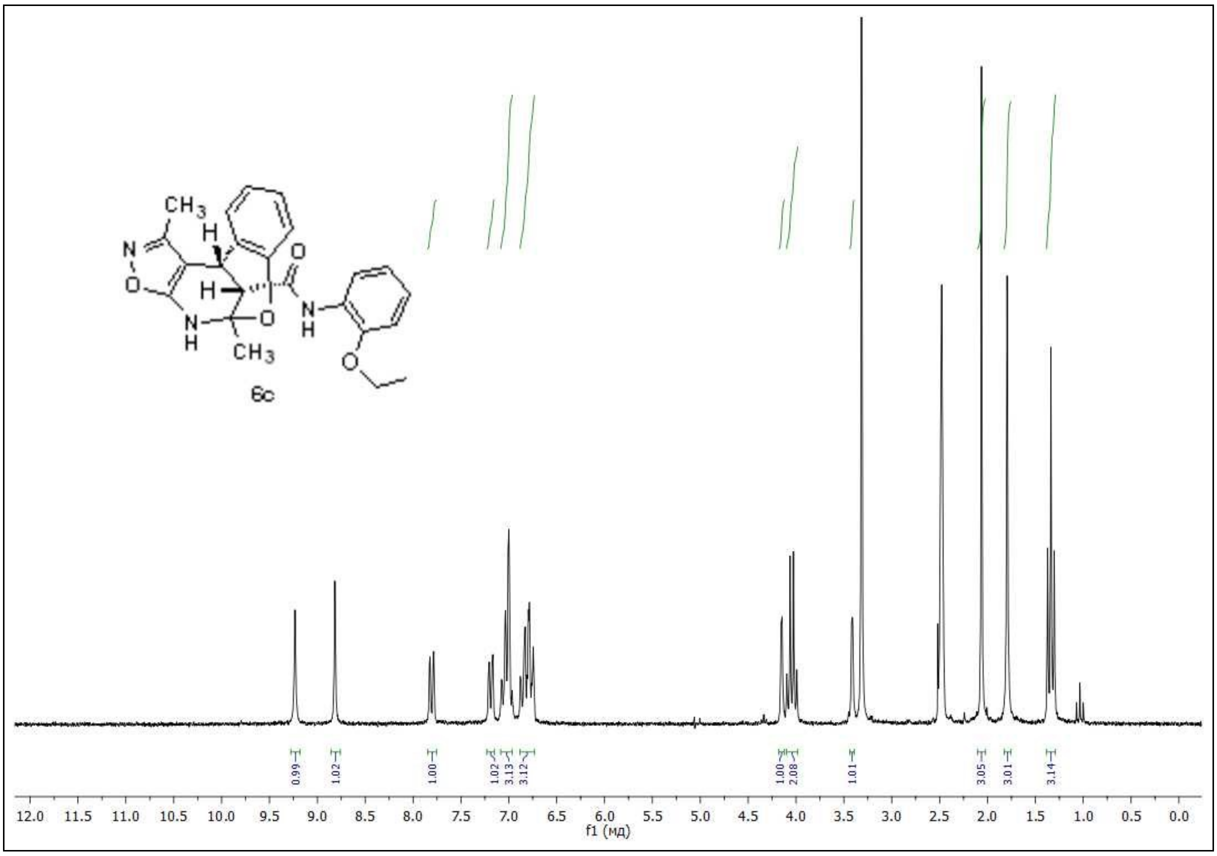

^{1}H NMR spectrum of compound **6c**

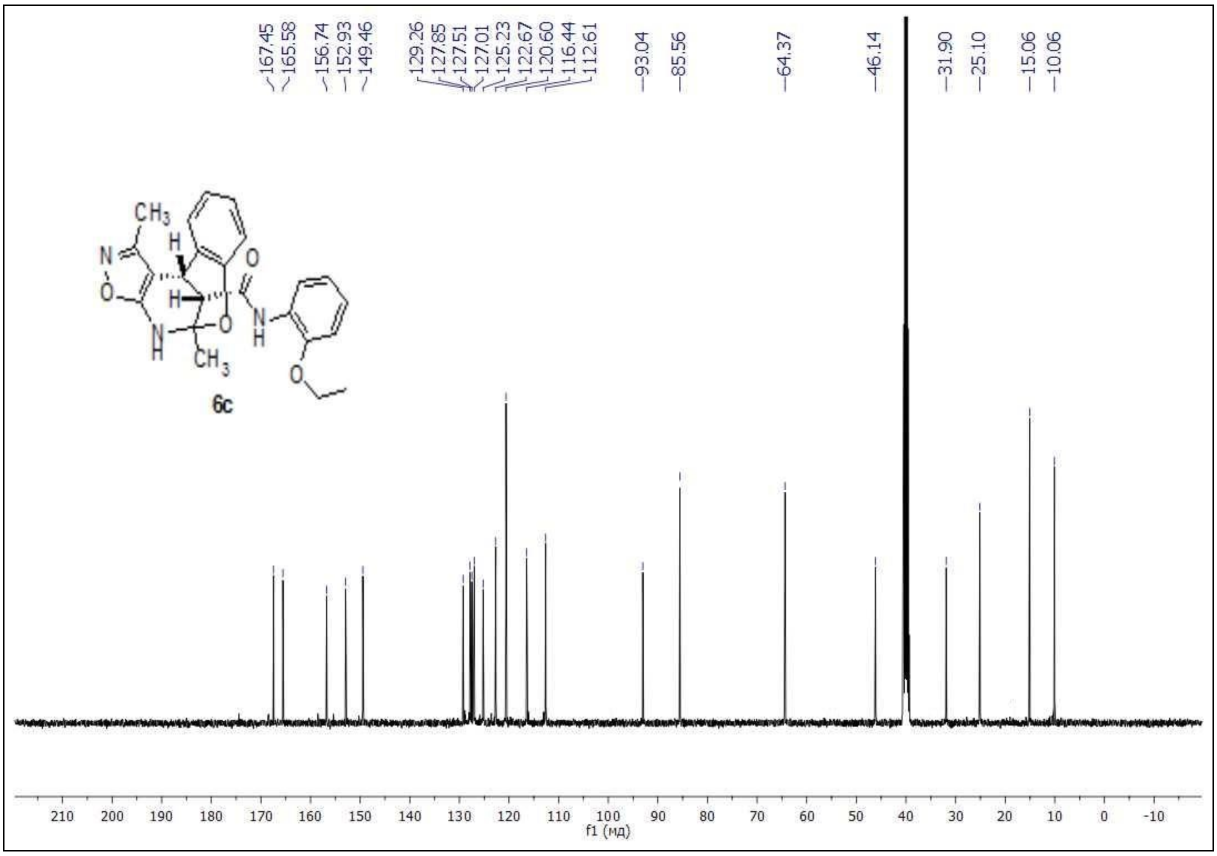

^{13}C NMR spectrum of compound **6c**

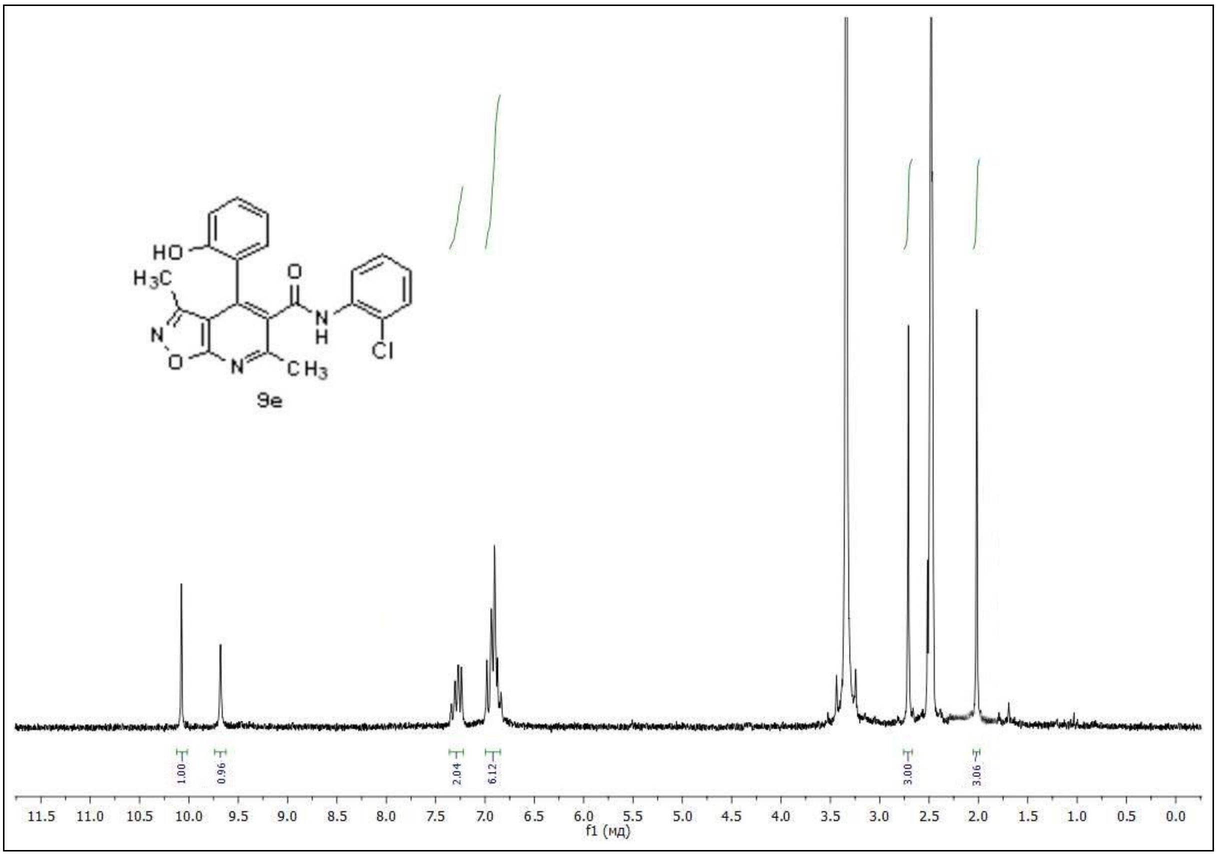

^{1}H NMR spectrum of compound **9e**

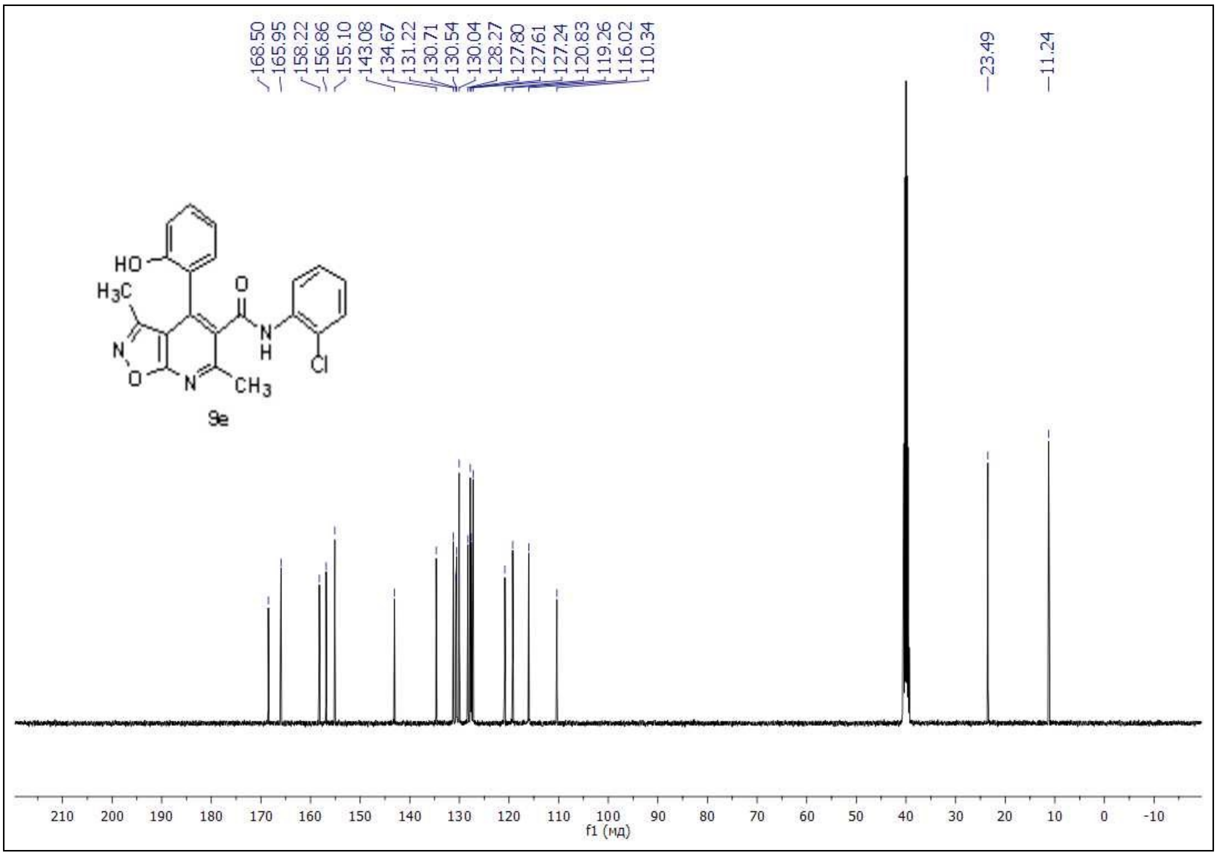

^{13}C NMR spectrum of compound **9e**

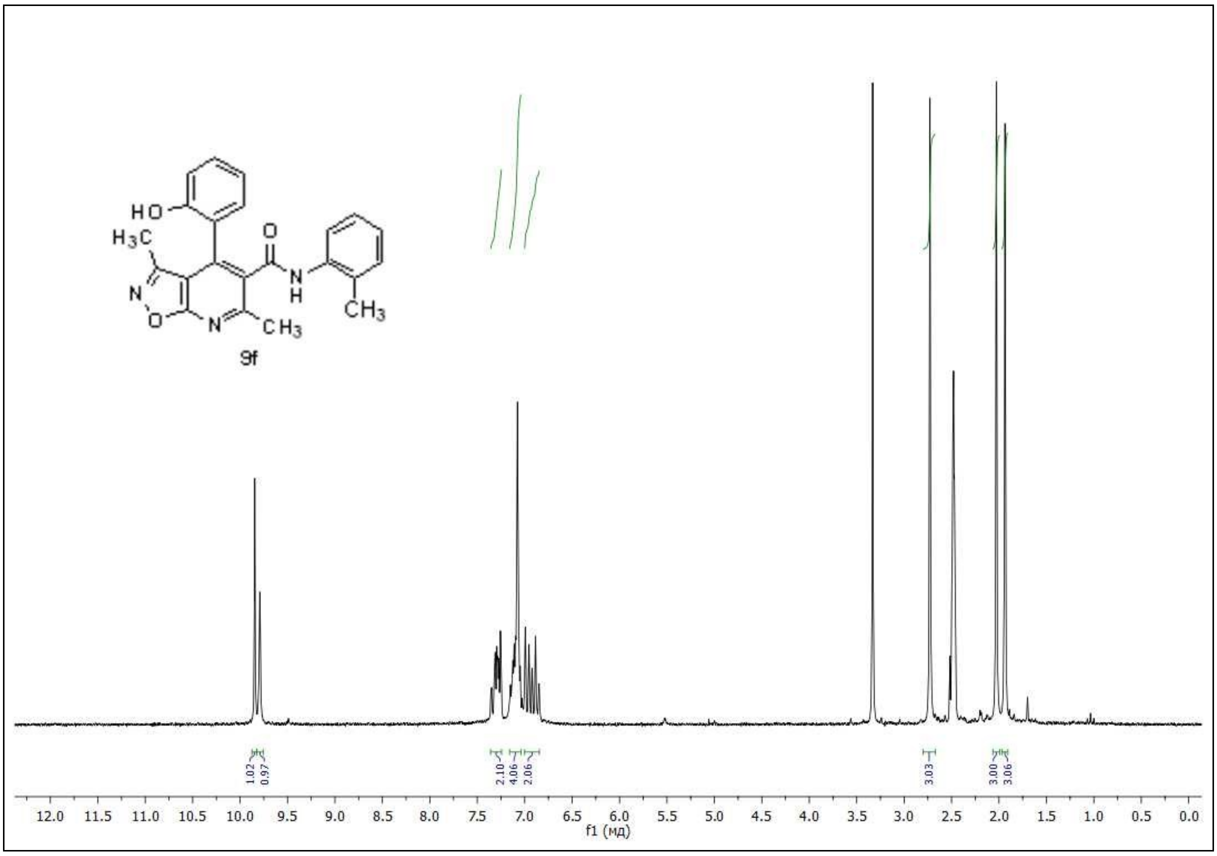

^{1}H NMR spectrum of compound **9f**

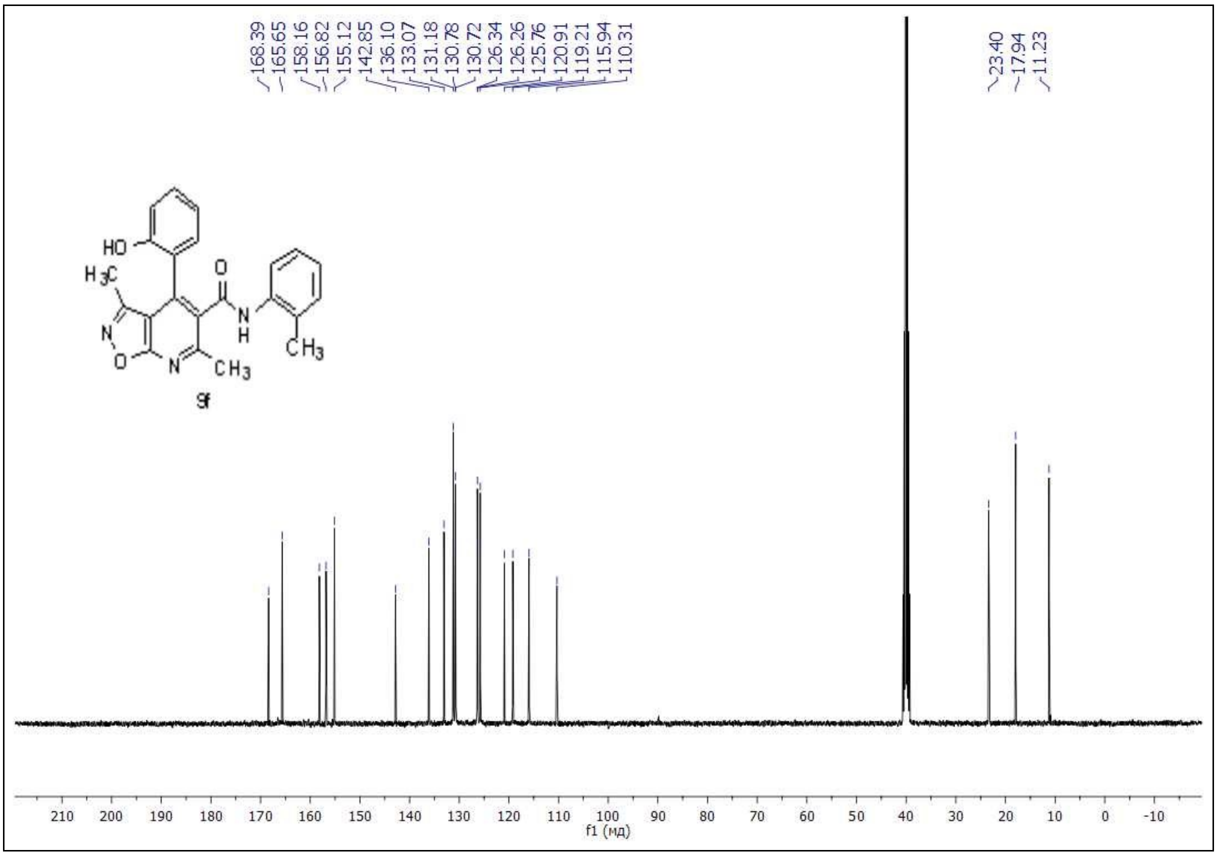

^{13}C NMR spectrum of compound **9f**

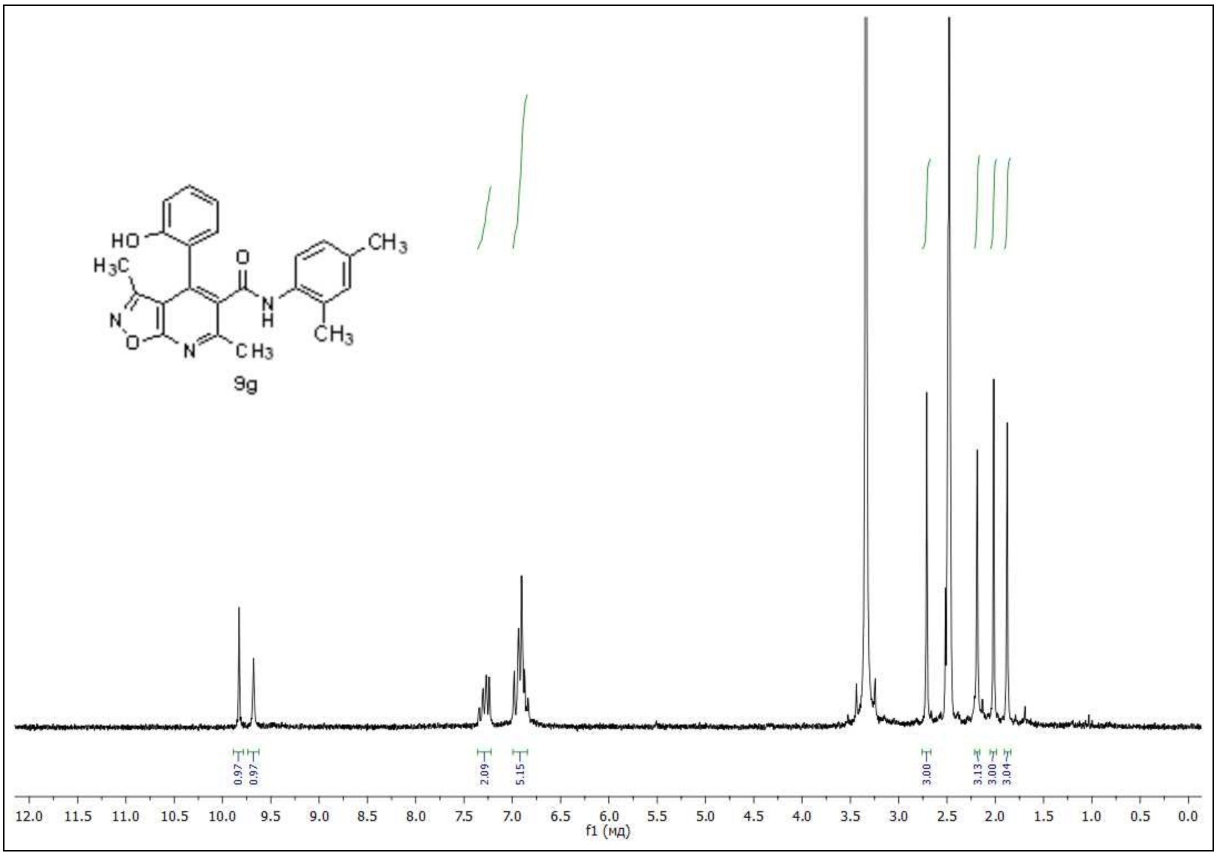

^{1}H NMR spectrum of compound **9g**

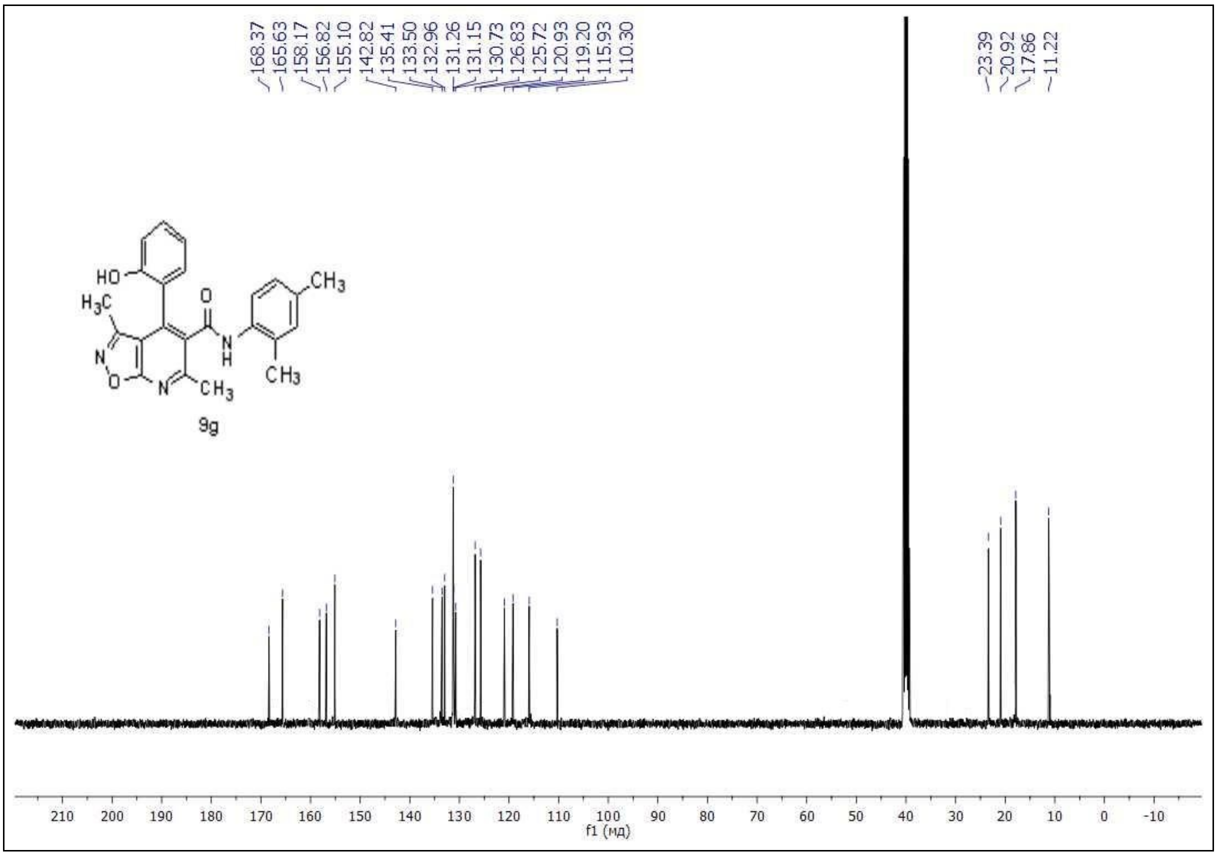

^{13}C NMR spectrum of compound **9g**
